# Amplification-free cancer diagnosis based on inhibition of Cas12a activity by site-specific 5mC-modified cfDNA

**DOI:** 10.1093/nar/gkaf1383

**Published:** 2025-12-17

**Authors:** Feng Yang, Can Xu, Chunhong Li, Xinni Xiang, Yi Zhao, Cong Hu, Huan Rong, Yu He, Jingyu Li, Yu Wang, Chao Tang, Xiaoyu Liu, Renyan Li, Fei Deng, Tingxiu Xiang

**Affiliations:** Chongqing Key Laboratory for the Mechanism and Intervention of Cancer Metastasis, Chongqing University Cancer Hospital, Chongqing University, Chongqing 400030, China; Institute of Image Processing and Pattern Recognition, Shanghai Jiao Tong University, 800 Dongchuan RD, Minhang District, Shanghai 200240, China; Chongqing Key Laboratory for the Mechanism and Intervention of Cancer Metastasis, Chongqing University Cancer Hospital, Chongqing University, Chongqing 400030, China; Chongqing Key Laboratory for the Mechanism and Intervention of Cancer Metastasis, Chongqing University Cancer Hospital, Chongqing University, Chongqing 400030, China; Chongqing Key Laboratory for the Mechanism and Intervention of Cancer Metastasis, Chongqing University Cancer Hospital, Chongqing University, Chongqing 400030, China; Chongqing Key Laboratory for the Mechanism and Intervention of Cancer Metastasis, Chongqing University Cancer Hospital, Chongqing University, Chongqing 400030, China; Chongqing Key Laboratory for the Mechanism and Intervention of Cancer Metastasis, Chongqing University Cancer Hospital, Chongqing University, Chongqing 400030, China; Chongqing Key Laboratory for the Mechanism and Intervention of Cancer Metastasis, Chongqing University Cancer Hospital, Chongqing University, Chongqing 400030, China; Department of Oncology, The First Affiliated Hospital of Chongqing Medical University, Chongqing 400016, China; Chongqing Key Laboratory for the Mechanism and Intervention of Cancer Metastasis, Chongqing University Cancer Hospital, Chongqing University, Chongqing 400030, China; Chongqing Key Laboratory for the Mechanism and Intervention of Cancer Metastasis, Chongqing University Cancer Hospital, Chongqing University, Chongqing 400030, China; Chongqing Key Laboratory for the Mechanism and Intervention of Cancer Metastasis, Chongqing University Cancer Hospital, Chongqing University, Chongqing 400030, China; Chongqing Key Laboratory for the Mechanism and Intervention of Cancer Metastasis, Chongqing University Cancer Hospital, Chongqing University, Chongqing 400030, China; Graduate School of Biomedical Engineering, Faculty of Engineering, University of New South Wales, Sydney, NSW 2052, Australia; Chongqing Key Laboratory for the Mechanism and Intervention of Cancer Metastasis, Chongqing University Cancer Hospital, Chongqing University, Chongqing 400030, China

## Abstract

DNA methylation detection holds significant value for cancer diagnosis and recurrence monitoring. However, current methods are often time-consuming, costly, and necessitate specialized techniques. The CRISPR–Cas system, particularly Cas12a, presents a precise and user-friendly platform for disease diagnosis. We developed the CRISPR-Methylated DNA Detection Test (CRISPR-MeDNA Test), a Cas12a-based method for detecting methylation in plasma cell-free DNA (cfDNA). The results reveal that 5mC-modified DNA significantly suppresses the *trans*-cleavage activity of Cas12a, depending on the methylation site, number, and interval spacing. Simultaneously, methylation of the non-target strand (NTS) suppresses Cas12a activity more strongly than methylation of the target strand (TS), as the NTS plays a critical role in R-loop formation, which is essential for Cas12a cleavage target DNA. Mechanistically, 5mC-modified DNA was found to trigger conformational rearrangements in the Cas12a complex, as predicted by AlphaFold3 modeling and corroborated by FRET assays. Notably, the combination of Cas12a with multiplexed guide RNAs enables effective discrimination between cfDNA from healthy donors and cancer patients without the need for pre-amplification, based on the inhibitory effects of methylated DNA on the Cas12a *trans*-cleavage activity. This work provides a Cas12a-based detection for a rapid, cost-effective, low-complexity method for 5mC-modified cfDNA in liquid biopsies.

## Graphical Abstract

**Graphical Abstract:**
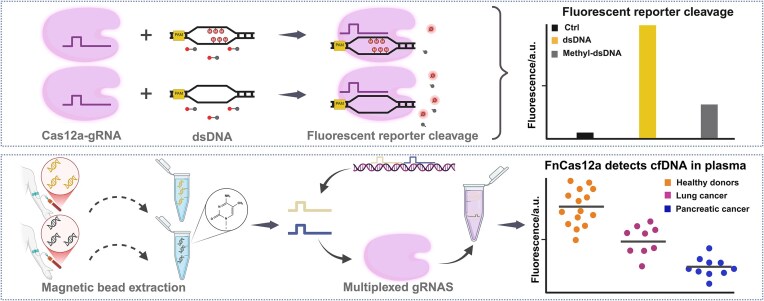

## Introduction

Epigenetics refers to heritable changes in gene expression that occur without alterations to the DNA sequence, contributing to phenotypic diversity [1]. DNA methylation, a key epigenetic modification referring to the addition of a methyl group to cytosine within CpG dinucleotides by DNA methyltransferases, plays crucial roles in regulating various biological processes [2, 3]. The predominant form of DNA methylation in mammals is 5-methylcytosine (5mC) [4]. It is reported that aberrant DNA methylation is closely associated with various diseases, particularly cancer [5–7]. Recent studies demonstrate that hypermethylation of cell-free DNA (cfDNA) serves as a biomarker for early cancer diagnosis and recurrence monitoring [8–13]. Consequently, various methods for detecting cfDNA methylation have emerged to facilitate cancer diagnosis.

Traditional methods for DNA methylation detection mainly include the bisulfite sequencing method based on chemical modification, the methylation-sensitive restriction endonuclease method based on enzymatic digestion, and the MBD protein and antibody enrichment method based on affinity enrichment, as well as sequencing-based methods such as reduced representation bisulfite sequencing and its enhanced whole-genome version. Each of these methods has its advantages and disadvantages, making them suitable for different research needs [14, 15]. Among them, bisulfite treatment is time-consuming and can lead to significant degradation and fragmentation of DNA [16–20]. The methylation-sensitive restriction endonuclease assay is limited by the specificity of DNA sequence and low sensitivity for methylation detection [21, 22]. Although the MBD enrichment method is effective for detecting methylated DNA, it is only sensitive to highly methylated genomic regions, which are prone to false negatives [15, 23–25]. In addition, methylation-specific sequencing is limited by complexity, cost, and expertise, restricting its clinical application [26, 27]. Therefore, developing precise, sensitive, rapid, and cost-effective approaches to detect abnormal cfDNA methylation is crucial for cancer diagnosis and recurrence monitoring.

CRISPR-based diagnostic tools have emerged as revolutionary molecular technologies due to their high sensitivity and accuracy [28–33]. CRISPR-associated enzymes, like Cas12a, can cleave specific double-stranded DNA (dsDNA) targets downstream of protospacer adjacent motifs (PAM) in *cis* [34] and subsequently exhibit non-specific single-stranded DNA (ssDNA) cleavage activity in *trans* [35–37]. This *trans*-cleavage property has been harnessed to produce detectable fluorescence signals for diagnostic purposes [29]. The CRISPR-Cas systems enable precise and personalized diagnosis through sequence-specific DNA cleavage [38]. Consequently, various CRISPR-Cas system-based biocatalytic signal amplification techniques have been developed for detecting DNA methylation [29], such as HOLMESv2 [39], DESCS [40], Cas12a-MSRE [41], CAM [42], DC-SDA [43], E-PfRPA/Cas [44], STABLE [45], meHOLMES [46], CHA [47], and HGRC [48], among others. Although Cas12a-based strategies have shown great potential for detecting methylated cfDNA, many current approaches still rely on bisulfite conversion or methylation-sensitive enzyme treatment before isothermal amplification, both of which are time-consuming and susceptible to false positives. Moreover, previous studies mainly focused on conventional DNA detection methods, which were not designed to identify patient-specific DNA methylation modifications. Therefore, deciphering how 5mC patterns modulate Cas12a activity is pivotal for developing amplification-free, Cas12a-based assays that can directly detect 5mC-bearing cfDNA in plasma.

In this study, we report for the first time that 5mC-modified DNA directly suppresses the *trans*-cleavage efficiency of Cas12a, primarily determined by the number and spacing of methylation sites. Based on this finding, we developed the CRISPR-Methylated DNA Detection Test (CRISPR-MeDNA Test), a Cas12a-based assay for detecting methylated plasma cfDNA for cancer diagnosis. The results showed that 5mC-modified cfDNA derived from cancer patients markedly inhibited Cas12a’s *trans*-cleavage activity, enabling rapid discrimination of cancer patients from healthy individuals within 30 min. Collectively, our findings establish a rapid, cost-effective, and low-complexity Cas12a-based liquid biopsy method for detecting 5mC-modified circulating tumor DNA (ctDNA).

## Materials and methods

### Reagents

EnGen^®^ Lba Cas12a protein was purchased from New England BioLabs Inc. (M0653T, NEB, USA). 10× NEBuffer r2.1 buffer was obtained from New England BioLabs Inc. (B6002S, NEB, USA). AsCas12a was procured from Genscript (Z03502, Genscript, China), and FnCas12a was purchased from Lixi Biosciences (LXC002L, Lixi, China). MgCl_2_ (M875550, Macklin, China) and KCl (R885888, Macklin, China) were used in the reaction solution. Dithiothreitol (DTT) was obtained from Thermo (R0861, Thermo Fisher Scientific, USA). DNase/RNase-free water was supplied by Promega (MC119A, Promega, USA).

### Oligonucleotides

All DNA and RNA oligonucleotides were synthesized from Sangon Biotech Co., Ltd. (Shanghai, China). Fluorescently labeled reporter probes were synthesized from GENCEFE (Jiangsu, China). ssDNA oligonucleotides were dissolved in DNase/RNase-free water to a storage concentration of 100 μM. The dsDNA complex was formed by annealing TS and NTS strands at a 1:1 molar ratio to a storage concentration of 100 μM. Two types of hemimethylated dsDNA, with methylation on either the TS or the NTS, were prepared individually. The TS hemimethylated dsDNA was constructed by annealing a methylated TS strand with an unmethylated NTS strand at a molar ratio of 1:1. Meanwhile, the NTS hemimethylated construct was formed by annealing an unmethylated TS strand with a methylated NTS strand at 1:1. Both complexes were prepared at a storage concentration of 100 μM. All the annealed dsDNA was stored at −80°C until use. The gRNA is also dissolved in RNase-free water to a storage concentration of 20 µM and stored at −80°C. Detailed sequences for all oligonucleotides used in this study were presented in Supplementary Tables S1–S9.

### The *trans*-cleavage efficiency of Cas12a on 5-mC modified DNA

The total CRISPR–Cas12a *trans*-cleavage reaction volume was 100 μl per well, which contained 25 nM target DNA, 1 × NEBuffer r2.1 (50 mM NaCl, 10 mM Tris–HCl, 10 mM MgCl₂, 100 μg/ml recombinant albumin), 25 nM Cas12a protein nuclease, and 25 nM gRNA. Cas12a and gRNA were pre-incubated at 26°C for 30 min to allow ribonucleoprotein (RNP) complex formation. Then, 150 nM fluorescent ssDNA reporter (Texas Red-CCCCC-BHQ2) was added. Background fluorescence (excitation/emission: 570/615 nm) was recorded prior to initiating the reaction by adding the DNA trigger. Fluorescence intensity was monitored at 26°C every 10 min for 2 h using a BioTek Synergy H1 plate reader (Synergy H1, BioTek, USA).

### FRET assay

Sequence information for all Cy5-labeled DNA and Cy3-labeled gRNAs is provided in Supplementary Table S5. Both Cy5-labeled ssDNA and dsDNA (annealing Cy5-labeled ssDNA with unlabeled complementary strand) were constructed. Subsequently, 25 nM DNA oligonucleotide solution was added in Cas12a reaction mixture containing 25 nM Cy3-labeled gRNA and 25 nM Cas12a. Fluorescence resonance energy transfer (FRET) was measured using a plate reader (Spark, TECAN, Austria) with excitation at 520 nm. Emission was recorded at 565 nm for the donor fluorophore (Fd) and at 665 nm for the acceptor fluorophore (Fa). Fluorescence intensity was monitored at 26°C every 10 min for 2 h.

### Optimization of reaction conditions for Cas12a-based DNA methylation

Since Cas12a enzymatic activity exhibits concentration-dependent responses to Mg²⁺, K⁺, and DTT [49–51], the optimal reaction concentrations of Mg²⁺, K⁺, and DTT were investigated to enhance discrimination between methylated and unmethylated DNA in this study. Therefore, the pre-assembled reaction mixtures for the three Cas12a variants were supplemented with several concentrations of MgCl₂, KCl, or DTT. The MgCl₂ concentrations were 10 (the Mg²⁺ concentration in 1× NEBuffer r2.1), 25, 35, or 45 mM. KCl concentrations were 15, 45, or 65 mM. DTT concentrations were 5, 10, or 20 mM. The ssDNA or dsDNA was added to the prepared reaction mixture to trigger Cas12a activity. Fluorescence intensity was monitored at 26 °C every 10 min for 2 h using a BioTek Synergy H1 plate reader, with an excitation wavelength of 570 nm and an emission wavelength of 615 nm.

### Database and visualization software

All protein sequences and PDB parameters are from UniProt (https://www.uniprot.org/). Structural modeling of Cas12a–gRNA–dsDNA complexes was carried out using AlphaFold 3 (https://alphafoldserver.com/). The structural alignment and root mean square deviation (RMSD) analysis were performed using PyMOL (v2.6, Schrödinger, LLC). CpG islands in the CCDC140 promoter were predicted using EMBOSS CpGplot under standard criteria [52]: length ≥200 bp, GC content ≥50%, and observed/expected CpG ratio ≥0.6. These thresholds distinguish biologically relevant CpG islands from spurious CpG-dense regions. DNA methylation profiles were retrieved from the SMART (Shiny Methylation Analysis Resource Tool) platform (http://www.bioinfo-zs.com/smart/), a comprehensive resource integrating TCGA and GEO epigenomic datasets. This tool enables gene-specific methylation visualization and comparative analysis across samples.

### Bisulfite conversion and droplet digital PCR for DNA methylation analysis

Tissue DNA was extracted using the TIANamp Genomic DNA Kit (DP304-03, TIANGEN, China), quantified with a Qubit 4 Fluorometer (Thermo Fisher Scientific, USA), and diluted to 1 ng/μl with nuclease-free water. Bisulfite conversion was performed using the EZ DNA Methylation-Gold™ Kit (D5006, Zymo Research, USA). Briefly, 20 μl DNA was mixed with 130 μl CT Conversion Reagent and subjected to the following thermocycling protocol: 98°C for 10 min, 64°C for 2.5 h, and held at 4°C for 20 min. Bisulfite-treated DNA was purified according to the manufacturer’s protocol, including desulphonation and two washing steps, and eluted in 200 μl M-Elution Buffer. Eluted DNA was stored at −20°C until further use. Methylation-specific droplet digital PCR (ddPCR) was performed using the QX200 Droplet Digital PCR System (Bio-Rad, USA). Each reaction contained 10 μl of 2× ddPCR Supermix for Probes (no dUTP), 900 nM forward and reverse primers, 250 nM methylation-specific (FAM-labeled) or unmethylation-specific (VIC-labeled) probe, bisulfite-converted DNA, and nuclease-free water. Primer and probe sequences are listed in Supplementary Table S8. Droplets were generated using DG8 cartridges with droplet generation oil in the Bio-Rad Droplet Generator, transferred to a 96-well ddPCR plate, sealed with foil, and amplified in a C1000 Touch™ Thermal Cycler with the following program: 95°C for 10 min, 40 cycles of 94°C for 30 s and 60°C for 60 s, and 98°C for 10 min. After amplification, droplets were read using the QX200 Droplet Reader and analyzed with QuantaSoft software (Bio-Rad, USA). No-template control was used as the negative control, EpiScope^®^ Methylated HCT116 gDNA (Cat. 3522, Takara, China) as the positive control, and ACTB (β-actin) was used as the internal reference to normalize input DNA. Primer information is provided in Supplementary Table S9.

### MALDI-TOF based profiling of bisulfite-treated oligonucleotides

Tissue DNA extracted using the TIANamp Genomic DNA Kit (DP304-03, TIANGEN, China) was treated with the EZ DNA Methylation-Gold™ Kit (D5006, Zymo Research, USA) according to the manufacturer’s instructions. Two microliters of the reaction mixture were purified using Agena Resin Clean (8040, Agena Bioscience, USA) and spotted onto SpectroCHIP (10600F, Agena Bioscience, USA) arrays. Mass spectra were acquired on the Agena MassARRAY Analyzer 4 using matrix-assisted laser desorption/ionization-time of flight (MALDI-TOF) in negative ion mode, and data were processed using MassARRAY Typer software to determine nucleotide-specific modification status. Primer information is provided in Supplementary Table S9.

### LOD and VAF Evaluation of Spike-in cfDNA with Multiple gRNAs in Plasma

Multiple gRNAs were designed based on the CCDC140 gene sequence, and the corresponding synthetic complementary DNA oligonucleotides were prepared (Supplementary Table S8). The TS and NTS were annealed at a 1:1 molar ratio to generate unmethylated dsDNA and 5mC-modified dsDNA (100 μM). For preparation of spike-in plasma samples, 50 μl of each dsDNA was uniformly mixed with 450 μl DNA-free plasma (60 601 001, LDT-Bioscience, China). Then, the DNA was extracted using the BeyoMag™ Cell-Free DNA Isolation Kit (D0085S, Beyotime, China), eluted in 30 μl nuclease-free water, quantified using a Quantus fluorometer (Promega, USA), and the concentrations of unmethylated and methylated cfDNA were adjusted to be equivalent. FnCas12a (50 nM) and two gRNAs (50 nM each) were pre-incubated at 26°C for 30 min to assemble the RNP complex, followed by addition of the ssDNA fluorescent reporter (2.5 μM, Texas Red-CCCCC-BHQ2). The same Cas12a detection system was used for plasma background evaluation, LOD determination, and VAF analysis. For each reaction, FnCas12a (50 nM), two gRNAs (50 nM each), and the ssDNA reporter (2.5 μM) were assembled as described above, and 2 μl plasma or dsDNA samples were then added, followed by adjusting the reaction mixture to a final volume of 20 μl. For LOD measurements, cfDNA was added at final concentrations of 1nM, 100 pM, 10 pM, 1 pM, 100 fM, and 10 fM. For VAF analysis, the total cfDNA concentration was 1 nM, spiking unmethylated DNA with varying proportions of methylated DNA (0%, 1%, 5%, 20%, 50%, and 100%). All reactions were incubated at 26°C for 30 min, and fluorescence was recorded using a BioTek Synergy H1 microplate reader.

### FnCas12a-based for detection of human methylation cfDNA in plasma

Human cfDNA was extracted from 4 ml of plasma from healthy individuals and cancer patients using the BeyoMag™ Cell-free DNA Isolation Kit (D0085S, Beyotime, China) according to the manufacturer’s instructions. cfDNA was eluted in 30 μl of DNase/RNase-free water and quantified using a Quantus Fluorometer (Quantus, Promega, USA). For detection of cfDNA methylation, the FnCas12a reaction mixture containing 50 nM FnCas12a and 50 nM of two gRNAs was incubated at 26°C for 30 min, followed by addition of 2.5 μM ssDNA reporter (Texas Red-CCCCC-BHQ2). The 384 plate was briefly centrifuged at 1200 rpm for 1 min at 4°C to collect the reaction mixture at the bottom of each well. Subsequently, 2 ng of cfDNA from healthy donors or patients with lung or pancreatic cancer was added to each well in a total reaction volume of 20 μl to activate Cas12a *trans*-cleavage. Fluorescence was measured after 30 min at 26°C using a BioTek Synergy H1 plate reader. All patient information is provided in Supplementary Table S10, Summary of clinical samples analyzed in this study. All clinical experiments were approved by the Ethics Committee of the Chongqing University Cancer Hospital (No. CZLS2022030-A-02), and informed consent was obtained from all patients prior to sample collection.

### Statistical analysis of data

All quantitative results are presented as the mean values derived from three independent experiments, with the standard error of the mean displayed as error bars. Statistical comparisons between two groups were performed using *t*-tests, while analyses involving multiple groups employed one-way ANOVA followed by appropriate post-hoc tests. (ns: non-significant; *P < .05; **P < .01; ***P < .001)

## Results

### 5mC-modified DNA suppressed the *trans*-cleavage efficiency of Cas12a

**Figure 1.**
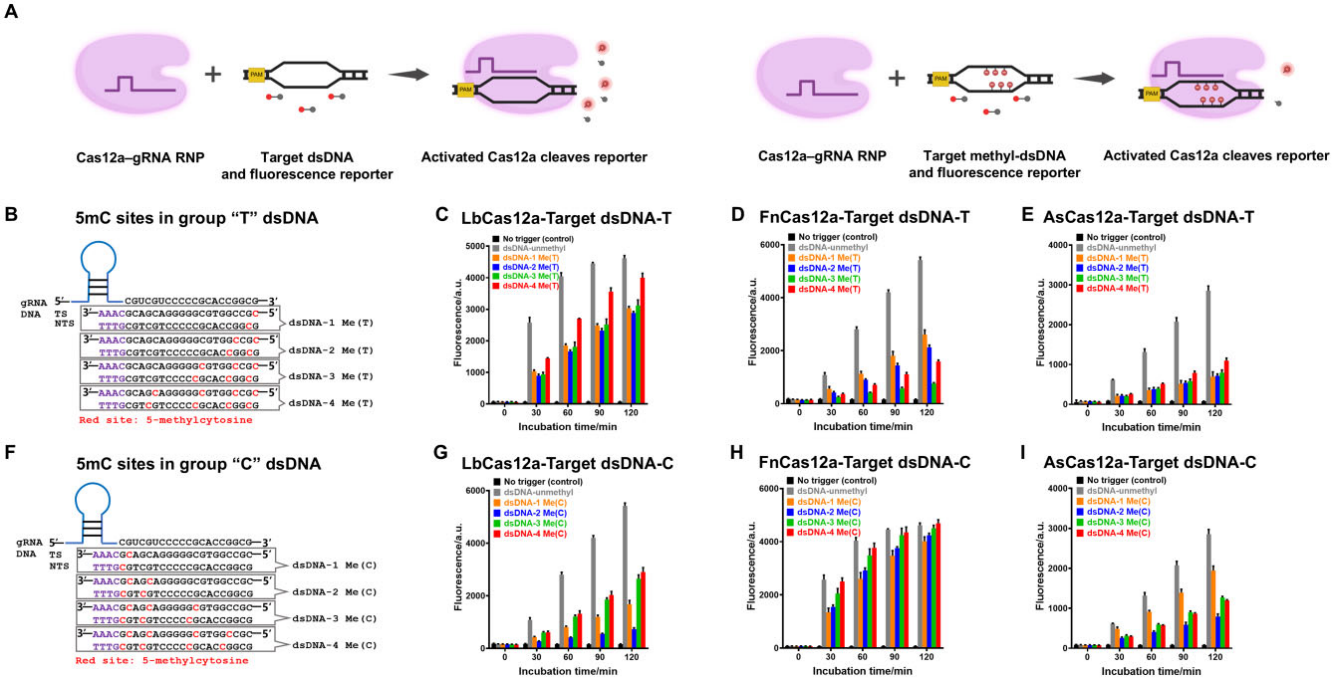
The different effects of 5mC-modified versus unmodified dsDNA on Cas12a *trans*-cleavage activity. (**A**) Schematic illustration of Cas12a–gRNA complex recognizing and cleaving either 5mC-modified or unmodified dsDNA, then triggering the *trans*-cleavage activity of Cas12a and being visualized by fluorescence intensity. Red cytosine residues indicate 5mC sites [created in BioRender. Deng, F. (2025) https://BioRender.com/qvpjyxs]. (**B**) Schematic of gRNA binding with dsDNA in group “T,” which contains 1–4 5mC modifications from the PAM-distal to the PAM-proximal region of the DNA. Red cytosine residues indicate 5mC sites. (C-E) The fluorescence intensity of LbCas12a (**C**), FnCas12a (**D**), and AsCas12a (**E**) was monitored at 0, 30, 60, 90, and 120 min upon triggering with group “T” dsDNA (*n *= 3). (**F**) Schematic of gRNA binding with dsDNA in group “C,” which contains 1–4 5mC modifications from the PAM-proximal to PAM-distal region along the TS. Red cytosine residues indicate 5mC sites. The fluorescence intensity was monitored at 0, 30, 60, 90, and 120 min for LbCas12a (**G**), FnCas12a (**H**), and AsCas12a (**I**) when triggered by group “C” dsDNA (*n* = 3).

To investigate the effect of 5mC-modified DNA on the *trans*-cleavage activity of CRISPR–Cas12a, we designed target DNA sequences with varying degrees of methylation. The CRISPR–Cas12a-based DNA detection system utilized 5mC-modified dsDNA or ssDNA as triggers (Fig. 1A and Supplementary Fig. S1A), each containing 1–4 5mC sites located either upstream or downstream of the PAM (TTTV) (Supplementary Table S1). Two groups of dsDNA substrates were designed based on the distribution pattern of 5mC modifications. In the “T” group, 5mC sites were introduced progressively from the PAM-distal toward the PAM-proximal region along the target strand (Fig. 1B). In the “C” group, modifications were arranged in the opposite direction from the PAM-proximal to the PAM-distal region (Fig. 1F). The same design principle was applied to the corresponding ssDNA substrates (Supplementary Fig. S1B and F).

We found that both when the ssDNA and dsDNA targets were modified with 5mC at different numbers and sites, it resulted in a decrease in fluorescence values compared to unmodified dsDNA and ssDNA (Fig. 1C–E, G–I and Supplementary Fig. S1C-E, G-I). However, the inhibitory effect of 5mC-modified DNA on Cas12a cleavage activity did not decrease linearly with increasing methylation sites. These results revealed no significant correlation between Cas12a *trans*-cleavage activity and the number of 5mC modifications on the target DNA.

### Sensitivity of single and interval 5mC modifications on Cas12a activity

**Figure 2.**
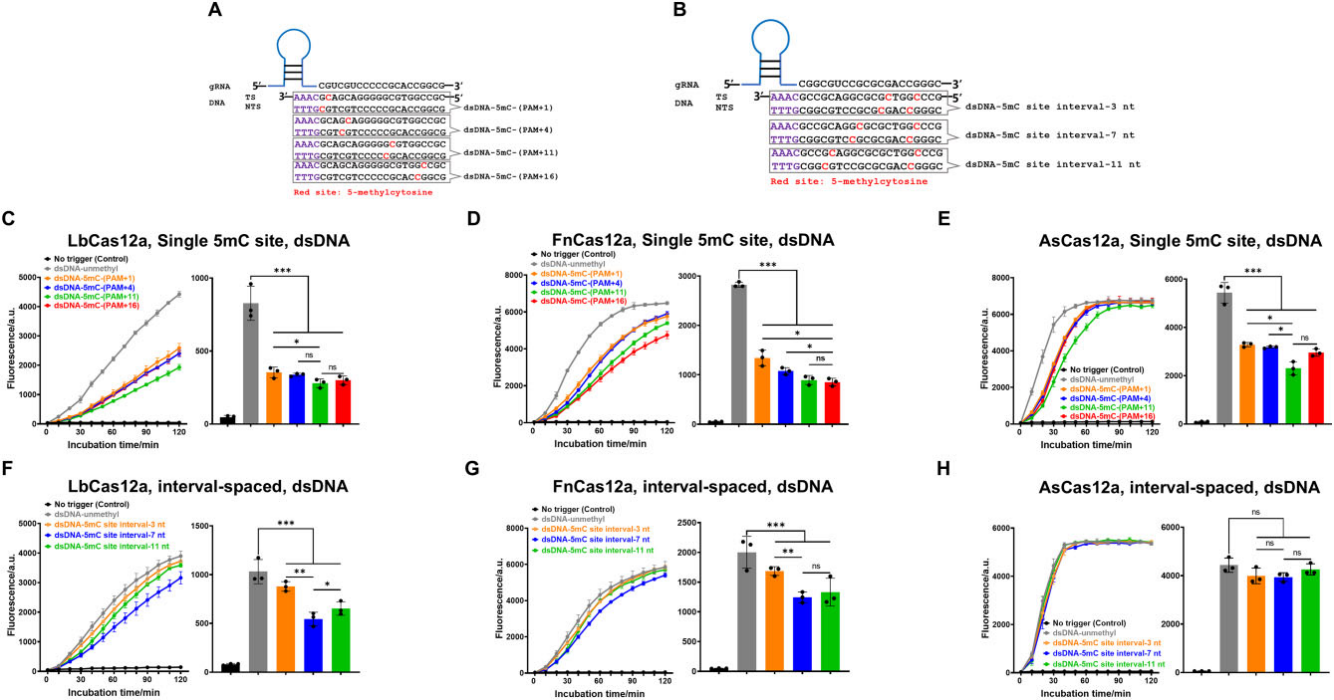
Sensitivity of dsDNA with single 5mC site and interval-spaced 5mC modifications to Cas12a activity. (**A**) Schematic of the various single 5mC site modifications on dsDNA regions. Red cytosine residues indicate 5mC. (**B**) Schematic of the 5mC modifications with different interval spaced on dsDNA. Red cytosine residues indicate 5mC sites. (C–E) Effect of a single 5mC site modification within dsDNA on the *trans*-cleavage activity of Cas12a variants. Bar plots show fluorescence intensity at 30 min (*n* = 3) for LbCas12a (**C**), FnCas12a (**D**), and AsCas12a (**E**). (ns: non-significant; *P < .05; ***P < .001). Effect of interval-spaced 5mC modifications within dsDNA on the *trans*-cleavage activity of Cas12a variants. Bar plots show fluorescence intensity at 30 min (*n* = 3) for LbCas12a (**F**), FnCas12a (**G**), and AsCas12a (**H**). (ns: non-significant; *P < .05; **P < .01; ***P < .001).

Furthermore, we investigated how single-site and interval-spaced 5mC modifications within the target DNA affect the *trans*-cleavage activity of Cas12a variants. Single 5mC modifications were designed at various sites, ranging from the PAM-proximal to the PAM-distal region (Fig. 2A). Meanwhile, two 5mC sites spaced by different base numbers were constructed: one 5mC-modified base was fixed at PAM + 16 nt of the PAM-distal region, while the second was located at 3, 7, or 11 nt, progressively approaching the PAM (Fig. 2B). Similar to the design for ssDNA, 5mC modifications at single sites and at interval-spaced positions were introduced into the target ssDNA (Supplementary Fig. S2A and B). Since the fluorescence intensity triggered by unmethylated and methylated DNA reached a plateau across the three Cas12a variants after a 2-h reaction, the maximum reaction time was set at 2 h before the reaction was terminated, and significantly higher sensitivity was observed within the initial 30 min of the reaction. Accordingly, the fluorescence values measured at 30 min were selected for statistical analysis.

Across all three Cas12a variants, both ssDNA and dsDNA substrates containing a 5mC modification exhibited suppressed fluorescence signals relative to their unmethylated counterparts, with a stronger inhibitory effect observed in dsDNA. In dsDNA, FnCas12a showed a gradual decline in *trans*-cleavage activity as the 5mC site moved farther from the PAM (Fig. 2D), whereas LbCas12a and AsCas12a displayed only minor changes under the same modification gradient (Fig. 2C and E). This position-dependent inhibitory pattern, in which more distal 5mC sites yield reduced cleavage efficiency, is referred to hereafter as the “edge effect.” For ssDNA substrates, a similar but less pronounced positional trend was observed, where the *trans*-cleavage signals showed minor variation with the placement of the 5mC site (Supplementary Fig. S2C–E).

Subsequently, the *trans*-cleavage activity of the three Cas12a variants on ssDNA and dsDNA containing two spaced 5mC modifications was determined. In the target dsDNA, the fluorescence intensity of both LbCas12a and FnCas12a showed the greatest reduction when the two 5mC sites were spaced by 7 nt, compared to the unmethylated control (Fig. 2F and G), while AsCas12a appeared less sensitive to the spacing between 5mC sites (Fig. 2H). For target ssDNA, little variation in *trans*-cleavage efficiency was observed among targets with 3, 7, or 11 nt spacer bases, suggesting that the spacing between 5mC modifications has a limited effect on ssDNA recognition by Cas12a (Supplementary Fig. S2F-H).

### The effect of hemimethylated DNA on the *trans*-cleavage activity of Cas12a

**Figure 3.**
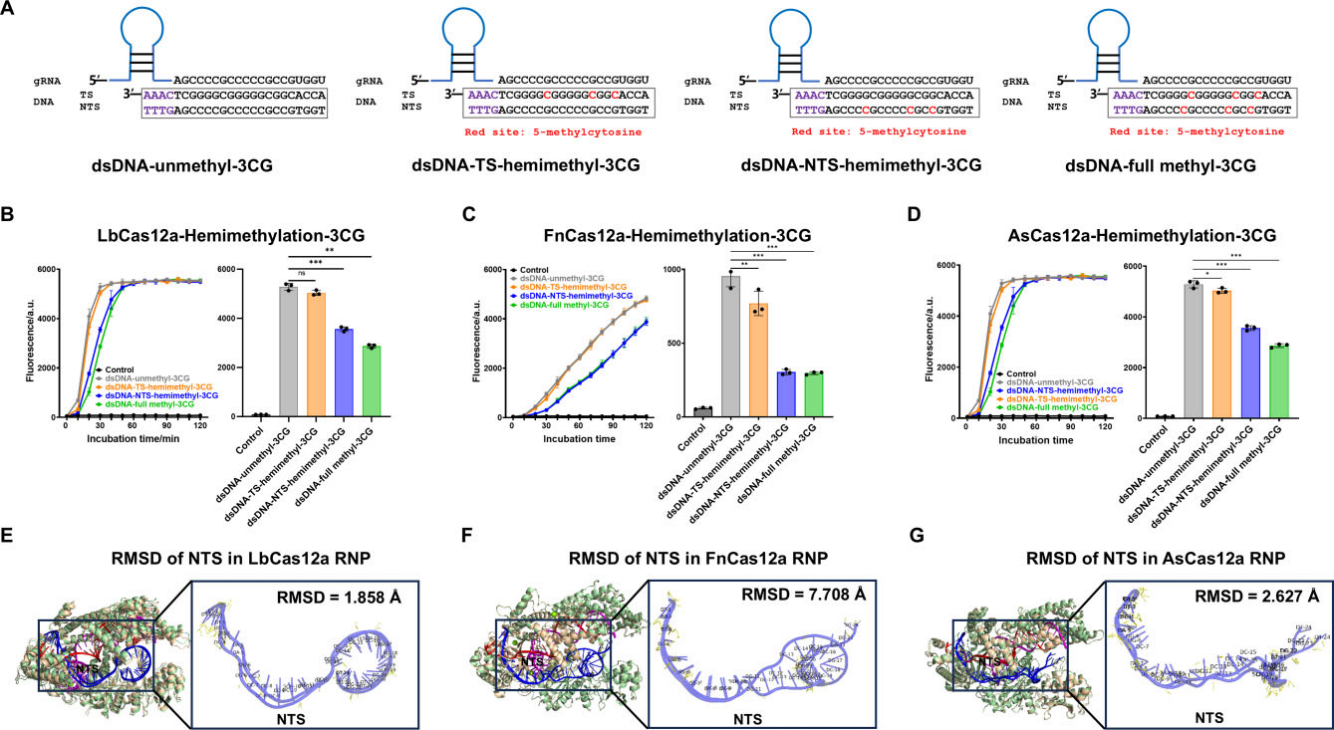
*Trans*-cleavage activity and structural conformation of Cas12a in response to hemimethylated dsDNA. (**A**) A schematic showing how gRNA binds to unmethylated, TS-hemimethylated, NTS-hemimethylated, and fully methylated dsDNA. Red cytosine residues indicate 5mC sites. Comparative analysis of the *trans*-cleavage activity of LbCas12a (**B**), FnCas12a (**C**), and AsCas12a (**D**), triggered by four types of 3CG target dsDNA: unmethylated, TS-hemimethylated, NTS-hemimethylated, and fully methylated dsDNA (*n *= 3). The column data showed the fluorescence intensity at 30 min of the reaction. (ns: non-significant; *P < .05; **P < .01; ***P < .001). The protein was shown in wheat (unmethylated dsDNA) and pale green (methylated dsDNA), with the TS in red, NTS in blue, and gRNA in magenta. The RMSD was 1.858 Å for LbCas12a (PDB: 5XU9) (**E**), 7.708 Å for FnCas12a (PDB: 6I1K) (**F**), and 2.627 Å for AsCas12a (PDB: 5b43) (**G**). The structure was enlarged to view the conformational change between the unmethylated NTS and the methylated NTS, based on RMSD analysis of three Cas12a variants.

As differentially hemimethylated regions in cfDNA represent potential independent biomarkers for cancer [53], we investigated whether hemimethylated dsDNA inhibits Cas12a *trans*-cleavage activity to a similar extent as methylated dsDNA. Two hemimethylated dsDNA groups were constructed: 3CG, containing three 5mC sites near the PAM-distal region, and 4CG, containing four 5mC sites located both near and distal to the PAM (Fig. 3A and Supplementary Fig. S3A). Each dsDNA type was prepared in four methylation states: unmethylated dsDNA, TS-hemimethylated dsDNA, NTS-hemimethylated dsDNA, and fully methylated dsDNA.

The results indicated that, compared to unmethylated DNA controls, methylated dsDNA significantly inhibited the cleavage activity of all three Cas12a variants in the 3CG group (Fig. 3B–D). The NTS-hemimethylated dsDNA inhibited Cas12a cleavage activity significantly more than the TS-hemimethylated dsDNA. TS-hemimethylated dsDNA activity was only slightly lower than the unmethylated control, whereas NTS-hemimethylated dsDNA inhibition was similar to that of full methylated dsDNA. In contrast, 4CG constructs showed smaller differences among variants and methylation types (Supplementary Fig. S3B–D). Among them, FnCas12a exhibited greater discrimination than LbCas12a or AsCas12a in both 3CG and 4 CG groups.

### 5mC modified on non-target strand modulate Cas12a activity by altering complex conformation

In order to examine the potential structural basis for the effect of 5mC modification of DNA on Cas12a activity, the conformation of the Cas12a–gRNA–dsDNA ternary complex was modeled using AlphaFold3 and visualized in PyMOL. Structural alignment was performed by comparing only the methylated strand in TS- and NTS-hemimethylated dsDNAs with the corresponding unmethylated strand in fully unmethylated dsDNA. In both the 3CG and 4CG groups, the RMSD values showed differences. RMSD heatmaps further demonstrated that the NTS-hemimethylated dsDNA consistently exhibited higher RMSD values than the TS-hemimethylated dsDNA, indicating greater conformational alterations of the NTS strand within the Cas12a–gRNA–dsDNA ternary complex (Supplementary Fig. S4A and B). In the 3CG group, structural alignment of NTS-hemimethylated dsDNA to the corresponding unmethylated dsDNA yielded RMSD values of 1.858 Å for LbCas12a (PDB: 5XU9), 7.708 Å for FnCas12a (PDB: 6I1K), and 2.627 Å for AsCas12a (Fig. 3E–G). In the 4CG group, the corresponding RMSD values were 3.531 Å for LbCas12a, 1.973 Å for FnCas12a, and 5.232 Å for AsCas12a (Supplementary Fig. S3E–G).

Notably, in both 3CG and 4CG *trans*-cleavage assays, the NTS-hemimethylated dsDNA showed lower fluorescence intensity than the TS-hemimethylated counterpart. Structural modeling showed that the NTS-hemimethylated dsDNA exhibited greater conformational deviation than TS-hemimethylated dsDNA within the Cas12a–gRNA–dsDNA ternary complex. Meanwhile, the fluorescence difference between unmethylated and methylated dsDNA was greater in the 3CG group than in the 4CG group. Together, these findings indicate that NTS hemimethylation more strongly suppresses the *trans*-cleavage activity of Cas12a than TS hemimethylation.

### The effect of 5mC modification on the structural dynamics of the Cas12a complex

**Figure 4.**
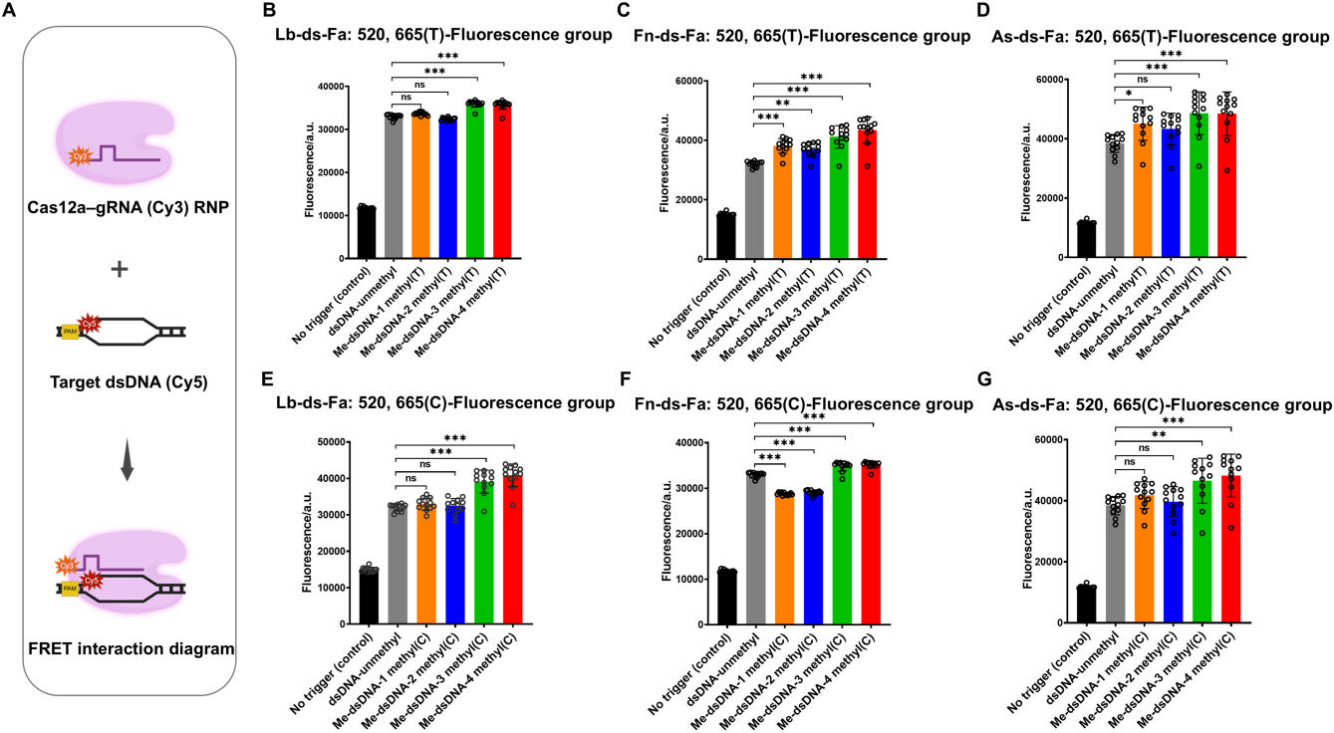
The conformation of 5mC-modified or unmodified dsDNA with Cas12a–gRNA analyzed by FRET. (**A**) A schematic illustrating the FRET assay, which was used to detect energy transfer between the Cy3-labeled gRNA (donor fluorophore) and the Cy5-labeled target dsDNA (acceptor fluorophore) upon Cas12a binding. Excitation of the Cy3 donor causes non-radiative energy transfer to the nearby Cy5 acceptor. The resulting Cy5 emission indicates conformational changes in the Cas12a–RNP–dsDNA complex [created in BioRender. Deng, F. (2025) https://BioRender.com/y0xcs71]. Energy transfer from the Fd to the Fa was evaluated in the group “T” dsDNA with 1 to 4 sites of 5mC modification, using LbCas12a (**B**), FnCas12a (**C**), and AsCas12a (**D**) (*n *= 3). The plotted data represent Fa fluorescence values measured every 10 min over a total duration of 2 h. (ns: non-significant; *P < .05; **P < .01; ***P < .001). Energy transfer from the Fd to the Fa was evaluated in the group “C” dsDNA with 1 to 4 sites of 5mC modification, using LbCas12a (**E**), FnCas12a (**F**), and AsCas12a (**G**) (*n *= 3). The plotted data represent Fa fluorescence values measured every 10 min over a total duration of 2 h. (ns: non-significant; **P < .01; ***P < .001).

To further investigate conformational changes of the Cas12a ternary complex induced by methylated dsDNA or ssDNA, we designed a FRET-based assay as shown in Fig. 4A and Supplementary Fig. S5A [54–56]. Previous studies revealed that the stability of FRET pairs varies depending on their positions within the Cas12a RNP complex [57]. The combination of Cy3 at the 5′ end of the gRNA (donor) and Cy5 at the 3′ end of the target DNA (acceptor) prevents fluorophore detachment and ensures a stable FRET signal during Cas12a activation [51]. The sequence of target DNA was the same as Fig. 1B and F and Supplementary Fig. S1B and F. In the FRET assay, excitation of Cy3-labeled gRNA triggers resonance energy transfer to Cy5-labeled target DNA when probe-target distance falls within 10 nm, generating quantifiable Cy5 emission at 670 nm, and fluorescence intensity increases inversely with distance [51]. The fluorescence signals were dynamically monitored every 10 min by a microplate reader for a total of 2 h.

Significant variations in energy transfer efficiency was observed in both 5mC dsDNA (Fig. 4B–G) and ssDNA of three Cas12a variants (Supplementary Fig. S5B–G). Interestingly, energy transfer efficiency was significantly higher in 5mC-modified dsDNA compared to unmethylated dsDNA (Fig. 4B–G), but markedly lower in 5mC-modified ssDNA relative to unmethylated ssDNA (Supplementary Fig. S5B–G). Moreover, the FRET efficiency (Fa) varied among different Cas12a variants in response to target DNAs with different numbers of 5mC modifications. The opposing effects of 5mC modifications, reducing fluorescence in ssDNA while enhancing it in dsDNA, were observed consistently in all three Cas12a variants, although the extent of these effects varied among them. These results demonstrated that 5mC modifications significantly alter the structural dynamics of the Cas12a complex.

### Cas12a-based detection of 5mC modified cfDNA in plasma of clinical samples

**Figure 5.**
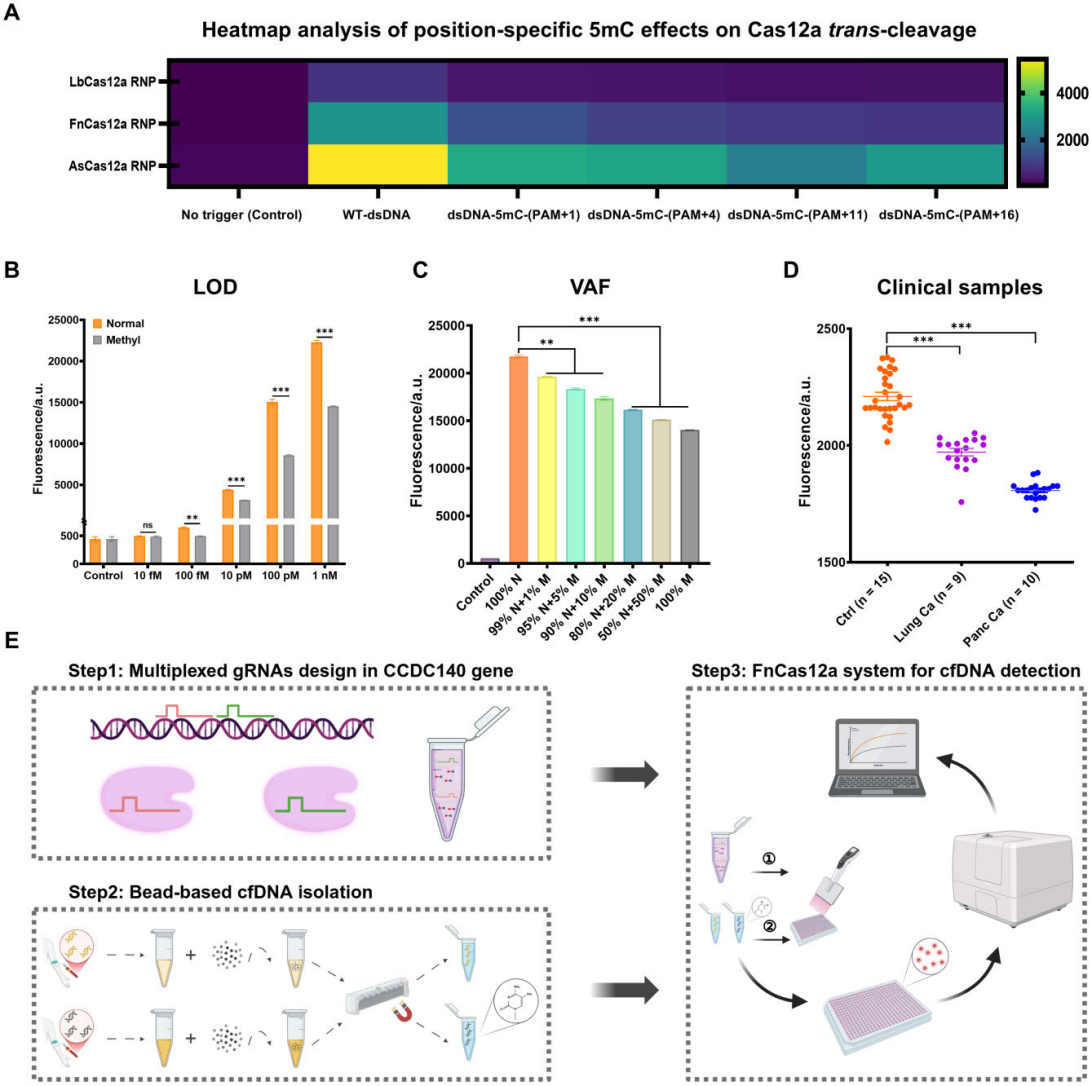
Discrimination of cfDNA from cancer patients and healthy donors based on Cas12a detection system. (**A**) Heatmap displays the *trans*-cleavage fluorescence intensity of LbCas12a RNP, FnCas12a RNP, and AsCas12a RNP in response to dsDNA containing a single 5mC modification at different sites. The fluorescence intensity monitored at 30 min after reaction, corresponding to the data shown in Fig. 2C–E. (**B**) LOD was determined using synthetic CCDC140 dsDNA corresponding to two gRNAs, unmethylated or methylated DNA, at 10 fM to 1 nM concentration (*n* = 2). (ns: non-significant; **P < .01; ***P < .001). (**C**) VAF analysis using synthetic CCDC140 dsDNA corresponding to multiple gRNAs, spiking unmethylated DNA with varying proportions of methylated DNA (0%, 1%, 5%, 20%, 50%, and 100%) (*n *= 2). (ns: non-significant; **P < .01; ***P < .001). (**D**) Fluorescence signals of Cas12a *trans*-cleavage activity triggered by cfDNA were measured in a preliminary cohort of 15 healthy donors (orange), 9 lung cancer patients (purple), and 10 pancreatic cancer patients (blue) (each sample in duplicate, *n* = 2). Fluorescence intensity was measured at 30 min. (ns: non-significant; ***P < .001). (**E**) Schematic illustration of the detection workflow for cfDNA from clinical samples using FnCas12a with a multi-gRNA strategy [created in BioRender. Deng, F. (2025) https://BioRender.com/j7gi1lw].

Plasma cfDNA methylation can serve as a tumor biomarker [58]. Given the pronounced inhibitory effect of methylated DNA on Cas12a’s cleavage activity, we hypothesized that Cas12a-based cfDNA detection could serve as a discriminative tool to differentiate cancer patients from healthy individuals. Firstly, the reaction system was optimized. Heatmap visualization of *trans*-cleavage results for three Cas12a variants targeting dsDNA with a single 5mC modification revealed that FnCas12a exhibited higher sensitivity than LbCas12a or AsCas12a (Fig. 5A). Therefore, FnCas12a was chosen for Cas12a-based detection of 5mC-modified cfDNA in clinical plasma samples.

Previous studies have demonstrated that metal ions, including magnesium (Mg²⁺) and potassium (K⁺), as well as the reducing agent DTT, can regulate the enzymatic activity of Cas12a [49–51]. Thus, the Cas12a *trans*-cleavage fluorescence reaction mixture (enzyme:gRNA:dsDNA = 25 :25 :25 nM) was supplemented with varying concentrations of Mg²⁺, K⁺, or DTT. A concentration gradient of MgCl₂ (10, 25, 35, and 45 mM) was applied, and 10 mM MgCl₂ corresponding to the concentration in 1× NEBuffer r2.1. KCl (15, 45, and 65 mM) or DTT (5, 10, and 20 mM) was also tested (Supplementary Fig. S6). At 30  min, the addition of Mg²⁺ increased the relative *trans*-cleavage fluorescence difference between methylated and unmethylated dsDNA from 59% to 84% (Supplementary Fig. S6C–F). Conversely, the addition of K⁺ diminished the differential signal from 74% to 58% (Supplementary Fig. S6G–I). Of the three DTT concentration groups tested, 10 mM yielded the greatest discrimination of 70% (Supplementary Fig. S6K). Thus, Mg²⁺, K⁺, and DTT levels modulate the Cas12a *trans*-cleavage activity between methylated and unmethylated synthetic dsDNA.

Based on preliminary results and facilitating clinical application, we next focused on the promoter region of the CCDC140 gene. The EMBOSS CpGplot software was used to predict CpG islands within this promoter. The results indicated the presence of two CpG islands in the region chr19:222298127–222298846, located at positions 51–260 and 369–662 (Supplementary Fig. S7A). Subsequently, we utilized the SMART software to analyze the methylation profiles of these CpG islands in public datasets. The analysis revealed that promoter methylation levels of CCDC140 in both lung adenocarcinoma (LUAD, *n*= 449) and lung squamous cell carcinoma (LUSC, *n* = 370) were significantly higher than those in adjacent normal lung tissues (*n *= 68) (Supplementary Fig. S7B). Similarly, pancreatic adenocarcinoma (PAAD, *n *= 184) exhibited markedly elevated promoter methylation levels relative to healthy controls (*n *= 10) (Supplementary Fig. S7C). These findings suggest that the CCDC140 promoter commonly undergoes aberrant hypermethylation across multiple cancer types. Furthermore, we used nucleic acid MALDI-TOF mass spectrometry to investigate methylation at individual sites within the CpG islands. Significant methylation was detected at multiple CpG sites in both lung cancer and pancreatic cancer (Supplementary Fig. S7D). This phenomenon was further confirmed by ddPCR (Supplementary Fig. S7E). ddPCR also showed significantly higher CCDC140 methylation in pancreatic cancer tissues compared to the corresponding adjacent non-tumor tissues. Low-level methylation was also detected in the adjacent tissues, indicating that CCDC140 methylation is closely associated with pancreatic cancer. Based on these results, we selected CCDC140 as the key target for studying 5mC modifications in cfDNA. According to the MALDI-TOF-observed methylation sites, we designed two gRNAs targeting PAM-distal CG-methylated sites (Supplementary Fig. S7D and Supplementary Tables S6 and S7), as illustrated in the schematic diagram (Fig. 5E).

We next evaluated the detection performance of a Cas12a system employing a single gRNA, which achieved a limit of detection (LOD) of 10 pM for discriminating methylated from unmethylated DNA (Supplementary Fig. S8A). Based on previous evidence that parallel use of multiple gRNAs [2, 3] can synergistically enhance the *trans*-cleavage kinetics of Cas12a and improve detection sensitivity [58], we introduced a two-gRNA design into the Cas12a system. Meanwhile, to better mimic the complex biological milieu of plasma, synthetic methylated and unmethylated DNA sequences were spiked into DNA-free human plasma [57, 59], followed by magnetic bead-based DNA extraction. With this optimized setup, the two-gRNA Cas12a system achieved an LOD of 100 fM, allowing clear discrimination between methylated and unmethylated DNA in plasma samples (Fig. 5B).

We further assessed the inhibitory effect of methylated DNA on Cas12a activity by spiking unmethylated DNA with graded proportions of methylated species (0%, 1%, 5%, 20%, 50%, and 100%). The results indicated that even 1% methylated DNA significantly suppressed Cas12a activity (Fig. 5C). In addition, DNA-free human plasma was included as a control to evaluate potential interference from DNA-free plasma components. This control induced only minimal Cas12a activation, comparable to the blank, demonstrating the specificity of the Cas12a-based assay and its potential utility in cancer diagnosis (Supplementary Fig. S8B). Finally, to validate Cas12a-based detection in clinical samples, cfDNA was extracted from plasma of 15 healthy donors, 9 lung cancer patients, and 10 pancreatic cancer patients using the magnetic bead method. Cas12a *trans*-cleavage activity was significantly higher in cfDNA from healthy individuals than in that from cancer patients (Fig. 5D). Collectively, these results demonstrate the potential of the CRISPR-MeDNA Test, a Cas12a-based platform for 5mC-dependent cfDNA detection in liquid biopsy applications.

## Discussion

This study first systematically revealed that 5mC-modified DNA suppresses Cas12a *trans*-cleavage activity, which is related to the methylation site, number, and spacer bases. It was also found that methylation of the NTS causes significantly stronger suppression than TS methylation, due to the conformational changes of the Cas12a complex. Meanwhile, the use of two gRNAs in the Cas12a system increased the sensitivity by 100-fold (from 10 pM with a single gRNA to 100 fM), enhancing its ability to discriminate between methylated and unmethylated DNA. Furthermore, even 1% methylated DNA spiking in unmethylated DNA significantly suppressed Cas12a activity. Most importantly, when Cas12a is combined with two gRNAs, it can effectively distinguish cfDNA from healthy donors and cancer patients within 30 min, according to the effect of 5mC modification on the Cas12a *trans*-cleavage activity. Therefore, this study provides a chance for rapid, cost-effective, low-complexity liquid biopsy for cancer diagnosis.

To mimic the hypermethylated state commonly observed in tumor-derived cfDNA, and considering that plasma cfDNA in cancer patients is predominantly dsDNA with detectable ssDNA, we evaluated both forms simultaneously [60, 61]. This inhibitory effect was less pronounced in ssDNA, likely because ssDNA may activate Cas12a through a more straightforward mechanism, bypassing the need for strand separation during target recognition, as required for dsDNA [62, 63]. A previous study has shown that methylation enhances dsDNA flexibility and alters its twist and roll [64, 65]. These changes may increase the difficulty of dsDNA unwinding, especially given that Cas12a is intrinsically inefficient in unwinding and requires conformational rearrangement of the RNP complex [66].

Notably, the inhibitory effect of 5mC-modified DNA on Cas12a cleavage activity was not linearly proportional to methylation sites. Therefore, the effects of 5mC-modified DNA at different sites or with interval-spaced modifications within the dsDNA on the *trans*-cleavage activity of Cas12a variants were determined. A single 5mC modification at various sites was designed to span from the PAM-proximal to the PAM-distal region. The results showed that, regardless of whether it was triggered by ssDNA or dsDNA, the fluorescence intensity decreased as the 5mC site approached the PAM-distal end. Interestingly, in dsDNA, the lowest *trans*-cleavage activity of FnCas12a was observed when the 5mC modification was located at PAM + 16 nt, which was termed the “edge effect.” This site-specific inhibition may be attributable to biophysical constraints required for optimal R-loop formation and DNA bending, as recent studies suggest that the conserved type V R-loop topology evolved under selective pressures to accommodate cleavage via a single RuvC active site [67]. 5mC modifications may facilitate this process by inducing DNA bending or structural destabilization at the PAM-distal region, thereby promoting target strand exposure and enabling conformational rearrangement of the RNP complex. These rearrangements may reduce the donor-acceptor distance between labeled domains, resulting in an enhanced FRET signal. However, our data confirm conformational changes of the RNP complex associated with decreased Cas12a activity, as reported in a previous study [33], but fail to quantify the extent of structural rearrangement or define its exact correlation with cleavage efficiency. Nonetheless, while these conformational rearrangements may enhance FRET signals locally, they do not necessarily translate into improved cleavage efficiency, particularly when DNA flexibility impedes R-loop initiation or strand separation.

Given that symmetrical and hemimethylation patterns of cfDNA have been identified as potential tumor biomarkers [53], the impact of hemimethylation on the NTS or TS of dsDNA on Cas12a cleavage efficiency was evaluated. The two hemimethylated dsDNA groups were designed: 3CG, containing three 5mC sites near the PAM-distal region, and 4CG, containing four 5mC sites located both near and distal to the PAM. Overall, the distinction in *trans*-cleavage activity between methylated and unmethylated DNA among the three Cas12a variants was more pronounced in the 3CG group compared to the 4CG group, particularly for FnCas12a. In both groups, hemimethylation on the NTS exhibited a stronger inhibitory effect on the *trans*-cleavage activity of all three Cas12a variants than TS hemimethylation. Notably, in the 4CG group, a more distinct difference in *trans*-cleavage activity was observed for AsCas12a at approximately 25 min into the reaction. Furthermore, in dsDNA contexts, excessive rigidity or improper bending induced by certain 5mC modifications may delay NTS dissociation [65, 66, 68–70], thereby restricting access to the *trans*-cleavage site despite FRET-detected conformational changes. Consequently, this impairs the conformational prerequisites for R-loop formation, which is essential for Cas12a *trans*-cleavage activation [33]. Taken together, these findings support the notion that DNA methylation induces conformational alterations prior to Cas12a *trans*-cleavage activation by hindering NTS binding to the *trans*-cleavage site of Cas12a.

To optimize the detection specificity for methylated cfDNA, we first systematically assessed the impact of Mg²⁺, K⁺, and DTT concentrations on the methylation-specific *trans*-cleavage activity of Cas12a. While 45 mM Mg²⁺ improved the discrimination between methylated and unmethylated synthetic DNA, K⁺ exhibited a biphasic effect: 15 and 45 mM enhanced activity, whereas concentrations above 65 mM reduced total fluorescence, likely due to decreased Cas12a enzymatic activity [50]. Among the tested DTT concentrations, 10 mM yielded the greatest discrimination. Although Mg²⁺, K⁺, and DTT improved the distinction between synthetic methylated and unmethylated DNA, they did not significantly enhance the fluorescence difference between cfDNA from healthy donors and pancreatic cancer patients. Therefore, these additives were excluded from clinical sample testing without affecting the conclusions based on synthetic DNA. Subsequently, to exclude the other factors that may inhibit Cas12a cleavage in complex biological systems, we performed a controlled experiment by spiking 5mC-methylated and unmethylated DNA into DNA-free human plasma. The results showed that methylated DNA significantly suppressed Cas12a activity compared to its unmethylated counterpart. Importantly, the DNA-free plasma itself did not activate Cas12a, demonstrating the specificity of the Cas12a-based assay.

Given that two to three gRNAs in parallel can synergistically enhance Cas12a’s detection sensitivity [71], two gRNAs targeting the CCDC140 promoter region were designed based on MALDI-TOF and ddPCR results in this study, and the detection sensitivity was improved from 10 pM with a single gRNA to 100 fM. Furthermore, using the two gRNAs Cas12a system, we found that as little as 1% methylated DNA induced significant suppression of Cas12a activity. In clinical samples, it can effectively differentiate cfDNA from healthy donors and cancer patients, based on the inhibition of 5mC modification on Cas12a *trans*-cleavage activity. However, cfDNA is minimal in healthy individuals, and the proportion of ctDNA in cfDNA ranges from 0.1% to 90%, presenting a major detection challenge [72, 73]. Furthermore, the scheme for detecting the inhibition of Cas12a by methylated DNA remains susceptible to false positives and poor quantification. In addition, Cas12a activity may also be influenced by other epigenetic modifications, such as 5hmC and 5fC [74–79]. Therefore, future efforts should focus on enhancing sensitivity and specificity. Relevant strategies may include signal amplification cascades, enzyme-assisted target conversion, tandem CRISPR systems capable of cooperative activation, methylation-directed target enrichment, and protein engineering of Cas12a.

In conclusion, this study developed the CRISPR-MeDNA Test for diagnosing cancer via detecting DNA methylation in plasma cfDNA. The results reveal that 5mC-modified DNA significantly suppresses the *trans*-cleavage activity of Cas12a, depending on the methylation site and interval spacing. The combination of Cas12a with multiplexed guide RNAs enables effective discrimination between cfDNA from healthy donors and cancer patients without the need for pre-amplification, based on the inhibitory effects of methylated DNA on the Cas12a *trans*-cleavage activity. This work provides a Cas12a-based detection for rapid, cost-effective, low-complexity method for 5mC-modified cfDNA in liquid biopsies.

## Supplementary Material

gkaf1383_Supplemental_File

## Data Availability

All data supporting the findings of this study are included within the manuscript and its supplementary materials. Additional raw data, including molecular dynamics simulation files, docking models, and FRET experimental data, are available from the corresponding author upon reasonable request.
