# Supplementary material for: Amplification-free cancer diagnosis based on inhibition of Cas12a activity by site-specific 5mC-modified cfDNA
Source: Nucleic Acids Res. 2025 Dec 17;53(22):gkaf1383. doi: 10.1093/nar/gkaf1383 (PMC12709189; doi:10.1093/nar/gkaf1383)
Supplement: gkaf1383_Supplemental_File [file gkaf1383_supplemental_file.pdf]

Supplementary Table 1. The DNA sequences with different number of 5-methylcytosine modification and their corresponding gRNA

Name	Oligo type	Sequence5'-3'	Modification
gRNA (Lb)	ssRNA	AAUUUCUACUAAGUGUAGAUCGUCGUCCC CCGCACCGGCG	N/A
gRNA (Fn)	ssRNA	AAUUUCUACUGUUGUAGAUCGUCGUCCC CCGCACCGGCG	N/A
gRNA (As)	ssRNA	AAUUUCUACUCUUGUAGAUCGUCGUCCCC CGCACCGGCG	N/A
Repoter	ssDNA	CCCCCC	5' Texas Red / 3' BHQ2
ssDNA-unmethyl	ssDNA	CGCCGGTGCGGGGGACGACGCAAA	N/A
ssDNA-1 Me (T)	ssDNA	/i5MedC/GCCGGTGCGGGGGACGACGCAAA	/i5MedC/
ssDNA-2 Me (T)	ssDNA	/i5MedC/GC/i5MedC/GGTGCGGGGGACGAC GCAAA	/i5MedC/
ssDNA-3 Me (T)	ssDNA	/i5MedC/GC/i5MedC/GGTG/i5MedC/GGGGG ACGACGCAAA	/i5MedC/
ssDNA-4 Me (T)	ssDNA	/i5MedC/GC/i5MedC/GGTG/i5MedC/GGGGG A/i5MedC/GACGCAAA	/i5MedC/
cDNA-1 Me (T)	ssDNA	TTTGCGTCGTCCCCCGCACCGG/i5MedC/G	/i5MedC/
cDNA-2 Me (T)	ssDNA	TTTGCGTCGTCCCCCGCAC/i5MedC/GG/i5M edC/G	/i5MedC/
cDNA-3 Me (T)	ssDNA	TTTGCGTCGTCCCC/i5MedC/GCAC/i5MedC/ GG/i5MedC/G	/i5MedC/
cDNA-4 Me (T)	ssDNA	TTTGCGT/i5MedC/GTCCCC/i5MedC/GCAC/i 5MedC/GG/i5MedC/G	/i5MedC/
cDNA-unmethyl	ssDNA	TTTGCGTCGTCCCCCGCACCGGCG	N/A
ssDNA-1 Me(C)	ssDNA	CGCCGGTGCGGGGGACGA/i5MedC/GCAAA	/i5MedC/
ssDNA-2 Me(C)	ssDNA	CGCCGGTGCGGGGGA/i5MedC/GA/i5MedC/ GCAAA	/i5MedC/
ssDNA-3 Me(C)	ssDNA	CGCCGGTG/i5MedC/GGGGGA/i5MedC/GA/i 5MedC/GCAAA	/i5MedC/
ssDNA-4 Me(C)	ssDNA	CGC/i5MedC/GGTG/i5MedC/GGGGGA/i5Med C/GA/i5MedC/GCAAA	/i5MedC/
cDNA-1 Me(C)	ssDNA	TTTG/i5MedC/GTCGTCCCCCGCACCGGCG	/i5MedC/
cDNA-2 Me(C)	ssDNA	TTTG/i5MedC/GT/i5MedC/GTCCCCCGCACC GGCG	/i5MedC/
cDNA-3 Me(C)	ssDNA	TTTG/i5MedC/GT/i5MedC/GTCCCC/i5MedC/ GCACCGGCG	/i5MedC/
cDNA-4 Me(C)	ssDNA	TTTG/i5MedC/GT/i5MedC/GTCCCC/i5MedC/ GCAC/i5MedC/GGCG	/i5MedC/

Supplementary Table 2. The DNA with a single 5-methylcytosine modification at different sites and its corresponding gRNA

Name	Oligo type	Sequence5’-3’	Modification
ssDNA-unmethyl	ssDNA	CGCCGGTGCGGGGGACGACGCAAA	N/A
Me-ssDNA-5mC-(PAM+1)	ssDNA	CGCCGTGCGGGGGACGA/i5MedC/GCAAA	/i5MedC/
Me-ssDNA-5mC-(PAM+4)	ssDNA	CGCCGGTGCGGGGGA/i5MedC/GACGCAAA	/i5MedC/
Me-ssDNA-5mC-(PAM+11)	ssDNA	CGCCGGTG/i5MedC/GGGGGACGACGCAAA	/i5MedC/
Me-ssDNA-5mC-(PAM+16)	ssDNA	CGC/i5MedC/GGTGCGGGGGACGACGCAAA	/i5MedC/
cDNA-unmethyl	ssDNA	TTTGCGTCGTCCCCCGCACCGGCG	N/A
Me-cDNA-5mC-(PAM+1)	ssDNA	TTTG/i5MedC/GTCGTCCCCCGCACCGGCG	/i5MedC/
Me-cDNA-5mC-(PAM+4)	ssDNA	TTTGCGT/i5MedC/GTCCCCCGCACCGGCG	/i5MedC/
Me-cDNA-5mC-(PAM+11)	ssDNA	TTTGCGTCGTCCCC/i5MedC/GCACCGGCG	/i5MedC/
Me-cDNA-5mC-(PAM+16)	ssDNA	TTTGCGTCGTCCCCCGCAC/i5MedC/GGCG	/i5MedC/

Supplementary Table 3. Interval-spaced 5mC sites on DNA and their corresponding gRNAs

Name	Oligo type	Sequence5'-3'	Modification
gRNA (Lb)-interval	ssRNA	AAUUUCUACUAAGUGUAGAUCGGCGUCCGCGCGACCGGGC	N/A
gRNA (Fn)-interval	ssRNA	AAUUUCUACUGUUGUAGAUCGGCGUCCGCGCGACCGGGC	N/A
gRNA (As)-interval	ssRNA	AAUUUCUACUCUUGUAGAUCGGCGUCCGCGCGACCGGGC	N/A
ssDNA-unmethyl-interval	ssDNA	GCCCGGTCGCGCGGACGCCGCAAA	N/A
ssDNA-5mC site interval-3 nt	ssDNA	GCC/i5MedC/GGT/i5MedC/GCGCGGACGCCGCAAA	/i5MedC/
ssDNA-5mC site interval-7 nt	ssDNA	GCC/i5MedC/GGTCGCG/i5MedC/GGACGCCGCAAA	/i5MedC/
ssDNA-5mC site interval-11 nt	ssDNA	GCC/i5MedC/GGTCGCGCGGA/i5MedC/GCCGCAAA	/i5MedC/
cDNA-unmethyl-interval	ssDNA	TTTGCGGCGTCCGCGCGACCGGGC	N/A
cDNA-5mC site interval-3 nt	ssDNA	TTTGCGGCGTCCGCG/i5MedC/GAC/i5MedC/GGGC	/i5MedC/
cDNA-5mC site interval-7 nt	ssDNA	TTTGCGGCGTC/i5MedC/GCGCGAC/i5MedC/GGGC	/i5MedC/
cDNA-5mC site interval-11 nt	ssDNA	TTTGCGG/i5MedC/GTCCGCGCGAC/i5MedC/GGGC	/i5MedC/

Supplementary Table 4. The 3CG and 4CG and their corresponding gRNA

Name	Oligo type	Sequence5'-3'	Modification
gRNA (Lb-3CG)	ssRNA	AAUUUCUACUAAGUGUAGAUAGCCCCGCCCCCGCCGUGGU	N/A
gRNA (Fn-3CG)	ssRNA	AAUUUCUACUGUUGUAGAUAGCCCCGCCCCCGCCGUGGU	N/A
gRNA (As-3CG)	ssRNA	AAUUUCUACUCUUGUAGAUAGCCCCGCCCCCGCCGUGGU	N/A
gRNA (Lb-4CG)	ssRNA	AAUUUCUACUAAGUGUAGAUCCGGGAACCGCGAGCAAUCG	N/A
gRNA (Fn-4CG)	ssRNA	AAUUUCUACUGUUGUAGAUCCGGGAACCGCGAGCAAUCG	N/A
gRNA (As-4CG)	ssRNA	AAUUUCUACUCUUGUAGAUCCGGGAACCGCGAGCAAUCG	N/A
ssDNA-unmethyl-3CG	ssDNA	ACCACGGCGGGGGCGGGGCTCAAA	N/A
ssDNA-no Methyl-4CG	ssDNA	CGATTGCTCGCGGTTCCCGGCAAA	N/A
ssDNA-Methyl-3CG	ssDNA	ACCA/i5MedC/GG/i5MedC/GGGGG/i5MedC/GGGGCTCAAA	/i5MedC/
ssDNA-Methyl-4CG	ssDNA	/i5MedC/GATTGCT/i5MedC/G/i5MedC/GGTTCC/i5MedC/GGCAAA	/i5MedC/
cDNA-no Methyl-3CG	ssDNA	TTTGAGCCCCGCCCCCGCCGTGGT	N/A
cDNA-unmethyl-4CG	ssDNA	TTTGCCGGGAACCGCGAGCAATCG	N/A
cDNA-Methyl-3CG	ssDNA	TTTGAGCCC/i5MedC/GCCCC/i5MedC/GC/i5MedC/GTGGT	/i5MedC/
cDNA-Methyl-4CG	ssDNA	TTTGC/i5MedC/GGGAAC/i5MedC/G/i5MedC/GAGCAAT/i5MedC/G	/i5MedC/

Supplementary Table 5. The FRET sequences and their corresponding gRNA

Name	Oligo type	Sequence5'-3'	Modification
gRNA (Lb)	ssRNA	AAUUUCUACUAAGUGUAGAUCGUCG UCCCCCGCACCGGCG	5'-Cy3
gRNA (Fn)	ssRNA	AAUUUCUACUGUUGUAGAUCGUCGU CCCCCGCACCGGCG	5'-Cy3
gRNA (As)	ssRNA	AAUUUCUACUCUUGUAGAUCGUCGU CCCCCGCACCGGCG	5'-Cy3
ssDNA-unmethyl	ssDNA	CGCCGGTGCGGGGGACGACGCAAA	3'-Cy5
Me-ssDNA-1methyl(T)	ssDNA	/i5MedC/GCCGGTGCGGGGGACGACG CAAA	3'-Cy5
Me-ssDNA-2methyl(T)	ssDNA	/i5MedC/GC/i5MedC/GGTGCGGGGGA CGACGCAAA	3'-Cy5
Me-ssDNA-3methyl(T)	ssDNA	/i5MedC/GC/i5MedC/GGTG/i5MedC/GG GGGACGACGCAAA	3'-Cy5
Me-ssDNA-4methyl(T)	ssDNA	/i5MedC/GC/i5MedC/GGTG/i5MedC/GG GGGA/i5MedC/GACGCAAA	3'-Cy5
cDNA-unmethyl	ssDNA	TTTGCGTCGTCCCCCGCACCGGCG	3'-Cy5
Me-cDNA-1methyl(T)	ssDNA	TTTGCGTCGTCCCCCGCACCGG/i5Me dC/G	3'-Cy5
Me-cDNA-2methyl(T)	ssDNA	TTTGCGTCGTCCCCCGCAC/i5MedC/G G/i5MedC/G	3'-Cy5
Me-cDNA-3methyl(T)	ssDNA	TTTGCGTCGTCCCC/i5MedC/GCAC/i5 MedC/GG/i5MedC/G	3'-Cy5
Me-cDNA-4methyl(T)	ssDNA	TTTGCGT/i5MedC/GTCCCC/i5MedC/G CAC/i5MedC/GG/i5MedC/G	3'-Cy5
Me-ssDNA-1methyl(C)	ssDNA	CGCCGGTGCGGGGGACGA/i5MedC/G CAAA	3'-Cy5
Me-ssDNA-2methyl(C)	ssDNA	CGCCGGTGCGGGGGA/i5MedC/GA/i5 MedC/GCAAA	3'-Cy5
Me-ssDNA-3methyl(C)	ssDNA	CGCCGGTG/i5MedC/GGGGGA/i5MedC /GA/i5MedC/GCAAA	3'- Cy5
Me-ssDNA-4methyl(C)	ssDNA	CGC/i5MedC/GGTG/i5MedC/GGGGGA/i 5MedC/GA/i5MedC/GCAAA	3'-Cy5
Me-cDNA-1methyl(C)	ssDNA	TTTG/i5MedC/GTCGTCCCCCGCACCG GCG	3'-Cy5
Me-cDNA-2methyl(C)	ssDNA	TTTG/i5MedC/GT/i5MedC/GTCCCCCG CACCGGCG	3'-Cy5
Me-cDNA-3methyl(C)	ssDNA	TTTG/i5MedC/GT/i5MedC/GTCCCC/i5M edC/GCACCGGCG	3'-Cy5
Me-cDNA-4methyl(C)	ssDNA	TTTG/i5MedC/GT/i5MedC/GTCCCC/i5M edC/GCAC/i5MedC/GGCG	3'-Cy5

Supplementary Table 6. Target DNA sequences corresponding to multiple gRNAs designed for the CCDC140 gene

```
222298127 TGAACTAACATATGTTTCACAAGTGTGGGCGCAGCCGGGACAATTTCGAGACAACTTCGA 222298186
222298187 GACAATTTCGAATGGACAAATTGCGGAGAAGTTGCTTCTGCCGCTCAGAAGCCGGTTCAC 222298246
222298247 CTCCTTCTCCACCGCGGCATTTCCAAAACAACAGGGACAAGTCTCCCCGGCTCGCCGCAG 222298306
222298307 GCCTGACCGCCCAGCTCCGCCAGGATTTGCAGAGAGCAGCGCGCTCCATTTGCAGAAAGG 222298366
222298367 AAATCGAGTAGGTCCTCGCCCCCGACTGGTGCTTCTTGGGGTGTGGGGTGCCCAGGGAAT 222298426
222298427 GGGCTTCCTGGAAGCACCAAAGGAGCCTGCGGAGCCTGGGGATGGGGTGAGGCAGCCGGT 222298486
222298487 CCCAGGCCCTGGGATCCAGGCGGCGCGCTGAGGCCCTCCCTTACCTTCCAGCGGGAACCC 222298546
222298547 GCTACGCGGGTAGTTCTGCCCCGGGCCCGGCCGCATCATCCTGGGCACAGCGCCGGCCAG 222298606
222298607 CGTGGTCATCCTGGGGGCAGCTTCGCTCGGAAATTATATCCAGGTGAAGGCGAAACGGAA 222298666
222298667 AGGCGAGTGCGGCGCGGATGACCCTCGGGAACTATCCGGAGCGTGGAGAGCCCCTCCCCA 222298726
222298727 AAACGGCTGGAGAGAGAGGGAGGGACGCGGGGAGGGGGGCTGTCGGTTCCTAGTCCAGAG 222298786
222298787 GCCGGAGCTGGAACCCGGGAAAGGGGAGGACGGGGAGGCCCCGGAGTCCAGGATCCCGAG 222298846
```

Red: CpG sites indicate potential 5-methylcytosine modification positions.
Yellow-shaded region: DNA sequence on the CCDC140 gene complementary to gRNA1.
Blue-shaded region: DNA sequence on the CCDC140 gene complementary to gRNA2.
Note: The yellow- and blue-shaded regions overlap at one thymine (T) base.

Supplementary Table 7. Multiple gRNAs were designed to target the CCDC140 gene

Name	Oligo type	Sequence5'-3'	Modification
gRNA 1	ssRNA	AAUUUCUACUGUUGUAGAUCAGA GAGCAGCGCGCUCCAU	N/A
gRNA 2	ssRNA	AAUUUCUACUGUUGUAGAUCAGA AAGGAAAUCGAGUAGG	N/A

Supplementary Table 8. Multiple gRNAs targeting CCDC140 DNA fragments

Name	Oligo type	Sequence5’-3’	Modification
WT-CCDC140-ssDNA	ssDNA	CCTACTCGATTTCCTTTCTGCAAATGGAGCGCGCTGCTCTCTGCAAA	N/A
Me-CCDC140-ssDNA	ssDNA	CCTACT/i5MedC/GATTTCCTTTCTGCAAATGGAG/i5MedC/G/i5MedC/GCTGCTCTCTGCAAA	N/A
WT-CCDC140-cDNA	ssDNA	TTTGCAGAGAGCAGCGCGCTCCATTTGCAGAAAGGAAATCGAGTAGG	N/A
Me-CCDC140-cDNA	ssDNA	TTTGCAGAGAGCAG/i5MedC/G/i5MedC/GCTCCATTTGCAGAAAGGAAAT/i5MedC/GAGTAGG	N/A

Supplementary Table 9. Primers used for MALDI-TOF and ddPCR assays

Name	Oligo type	Sequence5’-3’	Fragment size/modification
CCDC140F10	ssDNA	AGGAAGAGAGTAATATATGTTTTATAAGTGTGGG	525bp
CCDC140T7R	ssDNA	CAGTAATACGACTCACTATAGGGAGAAGGCT CCTTCACCTAAATATAATTTCC	
CCDC140-mF	ssDNA	GGATTTGTAGAGAGTAGCGC	131bp
CCDC140-mR	ssDNA	CGCAAACTCCTTTAATACTTCC	
CCDC140-mP	ssDNA	TCGAGTAGGTTTTCGTTTTCG	5'-FAM，3'-MGB
ACTB-mF	ssDNA	TGGTGATGGAGGAGGTTTAGTAAGT	133bp
ACTB-mR	ssDNA	AACCAATAAAACCTACTCCTCCCTTAA	
ACTB-mP	ssDNA	TTGTGTGTTGGGTGGTGGTT	5'-VIC，3'-MGB

Supplementary Table 10. Summary of clinical samples analyzed in this study

Sample (No.)	Patient (No.)	Age	Sex	MassARRAY	ddPCR	CRISPR-Cas12a
1	Pan Ca 1	50	F	tissue	tissue	Plasma
2	Pan Ca 2	68	F	tissue	tissue	Plasma
3	Pan Ca 3	61	F	tissue	tissue	Plasma
4	Pan Ca 4	64	M	tissue	tissue	Plasma
5	Pan Ca 5	62	M	tissue	tissue	Plasma
6	Pan Ca 6	61	M	tissue	tissue	Plasma
7	Pan Ca 7	66	F	tissue	tissue	Plasma
8	Pan Ca 8	59	M	NA	tissue	Plasma
9	Pan Ca 9	66	F	NA	tissue	Plasma
10	Pan Ca 10	60	M	NA	tissue	Plasma
11	Pan A1	50	F	NA	tissue	NA
12	Pan A2	68	F	NA	tissue	NA
13	Pan A3	61	F	NA	tissue	NA
14	Pan A4	64	M	NA	tissue	NA
15	Pan A5	62	M	NA	tissue	NA
16	Pan A6	61	M	NA	tissue	NA
17	Pan A7	66	F	NA	tissue	NA
18	Pan A8	59	M	NA	tissue	NA
19	Pan A9	66	F	NA	tissue	NA
20	Pan A10	60	M	NA	tissue	NA
21	Lung Ca 1	49	M	tissue	NA	Plasma
22	Lung Ca 2	61	F	tissue	NA	Plasma
23	Lung Ca 3	65	M	tissue	NA	Plasma
24	Lung Ca 4	58	M	tissue	NA	Plasma
25	Lung Ca 5	70	M	tissue	NA	Plasma
26	Lung Ca 6	67	M	tissue	NA	Plasma
27	Lung Ca 7	64	M	tissue	NA	Plasma
28	Lung Ca 8	70	F	tissue	NA	Plasma
29	Lung Ca 9	63	F	tissue	NA	Plasma
30	Lung A 1	49	M	tissue	NA	NA
31	Lung A 2	61	F	tissue	NA	NA
32	Lung A 3	65	M	tissue	NA	NA
33	Lung A 4	58	M	tissue	NA	NA
34	Lung A 5	70	M	tissue	NA	NA
35	Lung A 6	67	M	tissue	NA	NA
36	Lung A 7	64	M	tissue	NA	NA
37	Health1	46	M			Plasma
38	Health2	37	M			Plasma
39	Health3	33	M			Plasma
40	Health4	38	M			Plasma
41	Health5	29	F			Plasma
42	Health6	32	F			Plasma
43	Health7	35	F			Plasma
44	Health8	34	M			Plasma
45	Health9	54	F			Plasma
46	Health10	27	F			Plasma

47	Health11	28	F	Plasma
48	Health12	30	F	Plasma
49	Health13	27	F	Plasma
50	Health14	30	F	Plasma
51	Health15	29	M	Plasma

Note: F as female, M as male. N/A as no sample. Pan Ca as pancreatic cancer, Pan A as adjacent non-tumor tissue of pancreas. Lung Ca as lung cancer, Lung A as adjacent non-tumor tissue of lung.

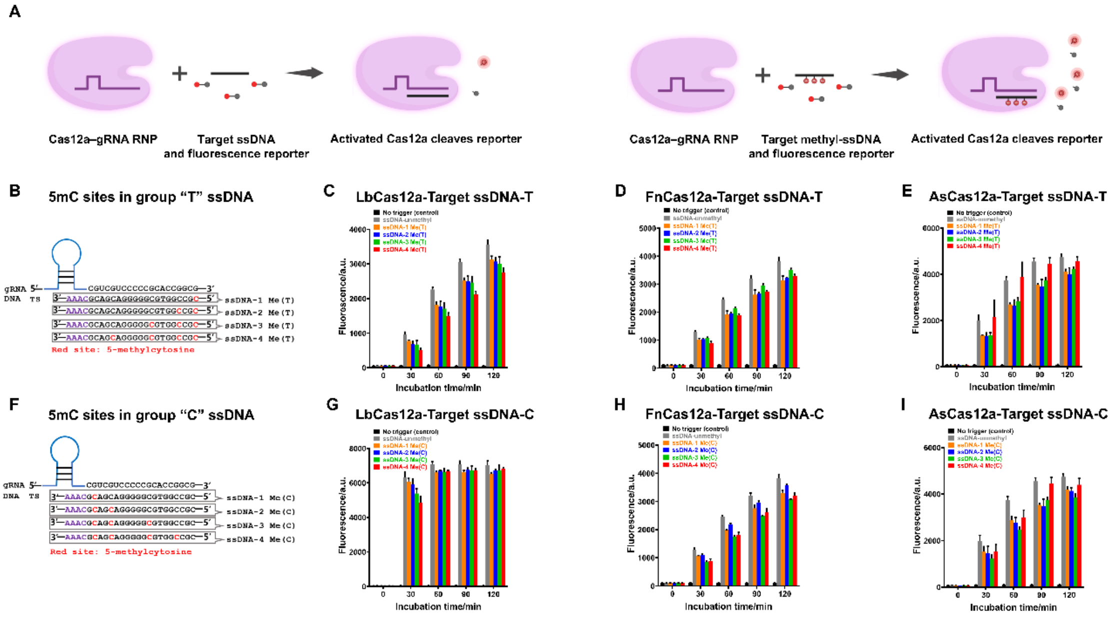

Supplementary Figure 1.

The different effects of 5mC-modified vs. unmodified ssDNA on Cas12a *trans*-cleavage activity

(A) Schematic illustration of Cas12a-gRNA complex recognizing and cleaving either 5mC-modified or unmodified ssDNA, then triggering the *trans*-cleavage activity of Cas12a and being visualized by fluorescence intensity. Red cytosine residues indicate 5mC sites (Created in BioRender. Deng, F. (2025) https://BioRender.com/8zgyw9e).

(B) Schematic of gRNA-ssDNA interactions in group “T,” with 1-4 5mC modifications progressively introduced from the 5′ to 3′ end of the target strand. Red cytosine residues indicate 5mC sites.

(C-E) The fluorescence intensity of LbCas12a (C), FnCas12a (D), and AsCas12a (E) was monitored at 0, 30, 60, 90, and 120 min upon triggering with group “T” ssDNA (n = 3).

(F) Schematic of gRNA-ssDNA interactions in group “C,” with 1-4 5mC modifications progressively introduced from the 3′ to 5′ end of the target strand. Red cytosine residues indicate 5mC sites.

(G-I) The fluorescence intensity was monitored at 0, 30, 60, 90, and 120 minutes for LbCas12a (G), FnCas12a (H), and AsCas12a (I) when triggered by group “C” ssDNA (n = 3)

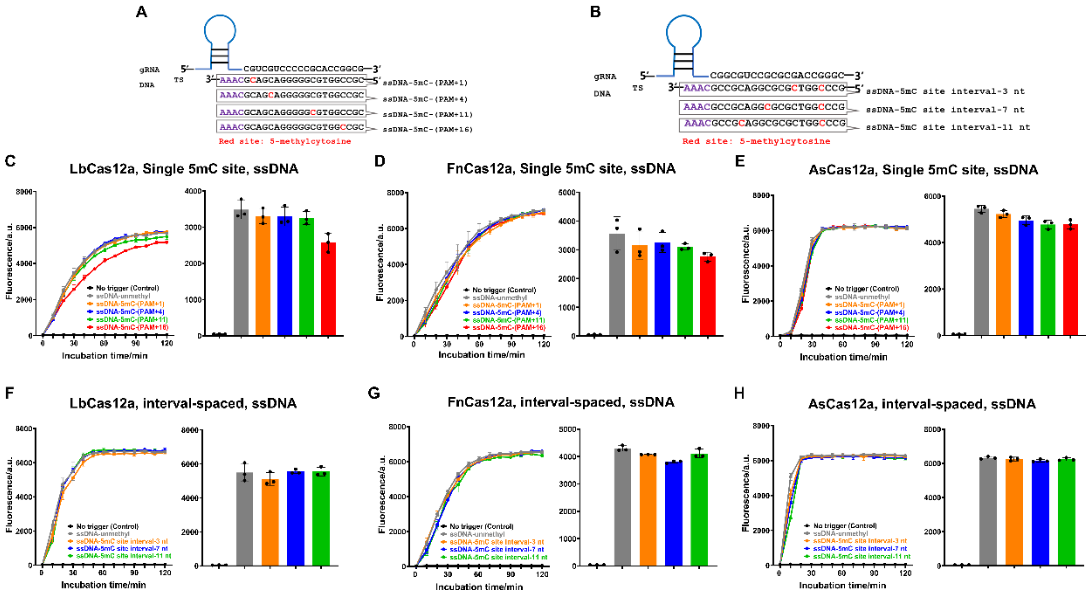

Supplementary Figure 2.

Sensitivity of ssDNA with single 5mC site and interval-spaced 5mC modifications to Cas12a activity

(A) Schematic of the various single 5mC site modifications on ssDNA regions. Red cytosine residues indicate 5mC.

(B) Schematic of the 5mC modifications with different interval-spaced on ssDNA. Red cytosine residues indicate 5mC sites.

(C-E) Effect of a single 5mC site modification within ssDNA on the *trans*-cleavage activity of Cas12a variants. Bar plots show fluorescence intensity at 30 minutes (n = 3) for LbCas12a (C), FnCas12a (D), and AsCas12a (E).

(F-H) Effect of interval-spaced 5mC modifications within ssDNA on the *trans*-cleavage activity of Cas12a variants. Bar plots show fluorescence intensity at 30 minutes (n = 3) for LbCas12a (F), FnCas12a (G), and AsCas12a (H).

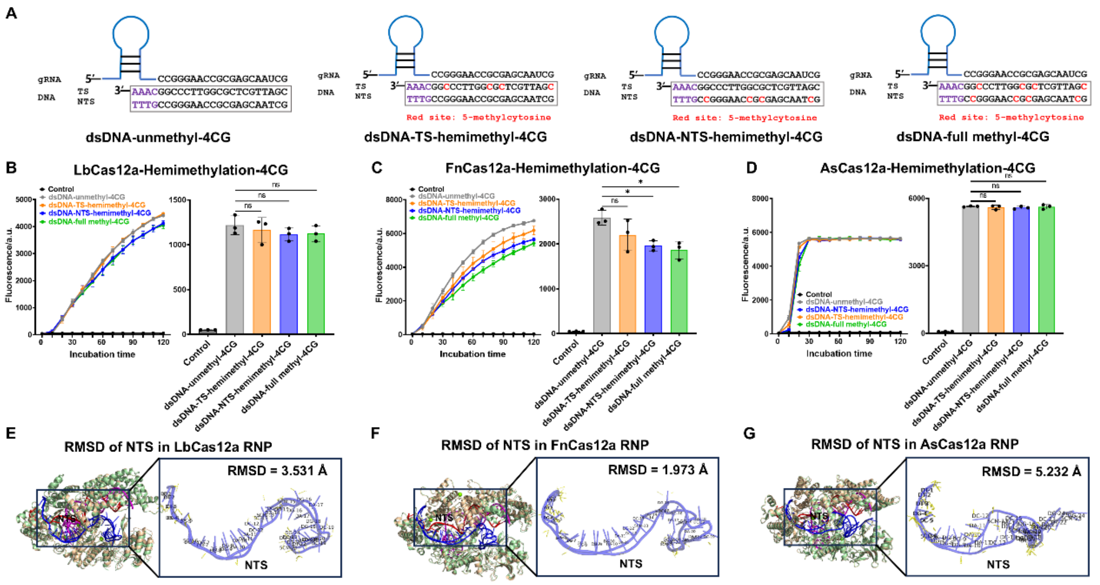

Supplementary Figure 3.

***Trans*-cleavage activity and structural conformation of Cas12a in response to hemimethylated dsDNA**

(A) A schematic showing how gRNA binds to unmethylated, TS-hemimethylated, NTS-hemimethylated, and fully methylated dsDNA. Red cytosine residues indicate 5mC sites.

(B-D) Comparative analysis of the *trans*-cleavage activity of LbCas12a (B), FnCas12a (C), and AsCas12a (D), triggered by four types of 4CG target dsDNA: unmethylated, TS-hemimethylated, NTS-hemimethylated, and fully methylated dsDNA (n = 3). The column data showed the fluorescence intensity at 30 min of the reaction. *(ns: non-significant; *p < 0.1).*

(E-G) The Cas12a nuclease was shown in wheat (unmethylated dsDNA) and palegreen (methylated dsDNA), with the TS in red, the NTS in blue, and the gRNA in magenta. The RMSD was 3.531 Å for LbCas12a (PDB: 5XU9), 1.973 Å for FnCas12a (PDB: 6I1K), and 5.232 Å for AsCas12a (PDB: 5b43). The structure was enlarged to view the conformational change between the unmethylated NTS and the methylated NTS, based on RMSD analysis of three Cas12a variants.

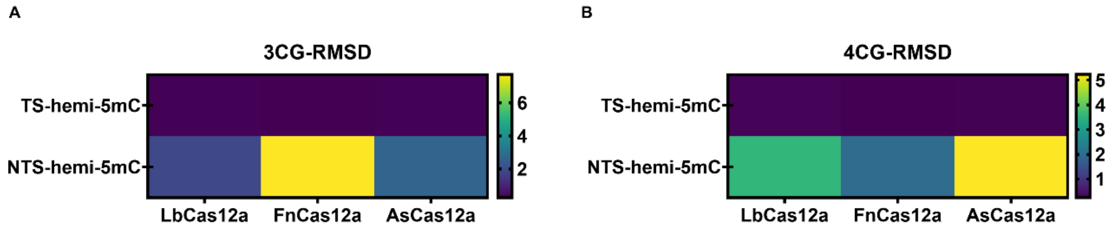

Supplementary Figure 4.

The RMSD values of Cas12a RNP complexes bound to hemimethylated and unmethylated dsDNA

(A) In the 3CG context, structural alignment of TS- and NTS-hemimethylated dsDNA with unmethylated dsDNA revealed RMSD differences indicative of strand-specific conformational changes.

(B) In the 4CG context, structural alignment of TS- and NTS-hemimethylated dsDNA with unmethylated dsDNA revealed RMSD differences indicative of strand-specific conformational changes.

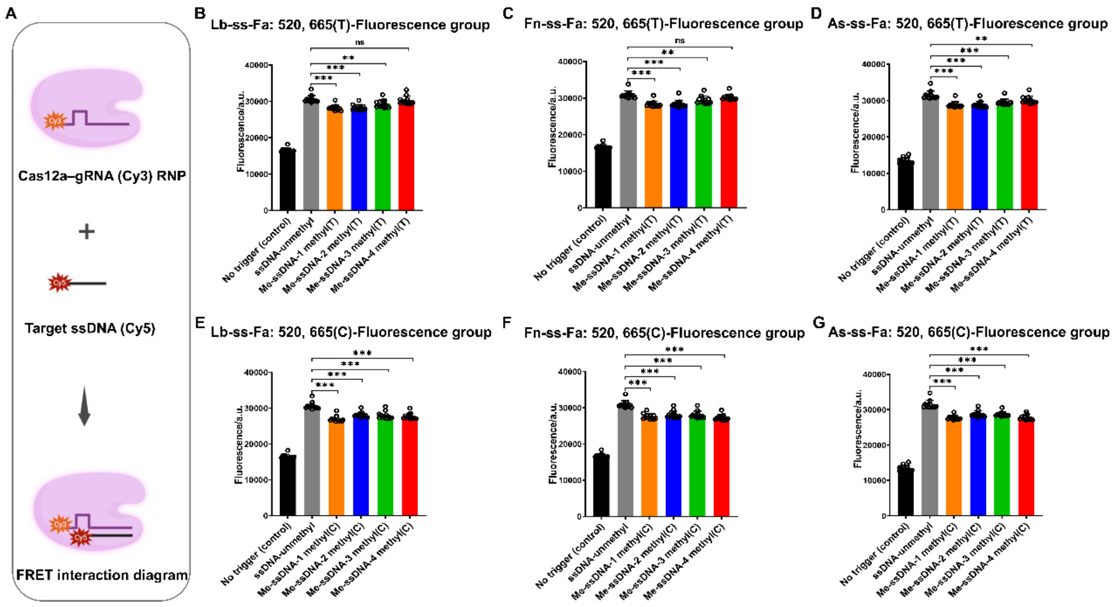

Supplementary Figure 5.

The conformation of 5mC-modified or unmodified ssDNA with Cas12a-gRNA analyzed by FRET

(A) A schematic illustrating the FRET assay, which was used to detect energy transfer between the Cy3-labeled gRNA (donor fluorophore) and the Cy5-labeled target ssDNA (acceptor fluorophore) upon Cas12a binding. Excitation of the Cy3 donor causes non-radiative energy transfer to the nearby Cy5 acceptor. The resulting Cy5 emission indicates conformational changes in the Cas12a-RNP-ssDNA complex (Created in BioRender. Deng, F. (2025) https://BioRender.com/52ex7ay).

(B-D) Energy transfer from the Fd to the Fa was evaluated in the group “T” ssDNA with 1 to 4 sites of 5mC modification, using LbCas12a (B), FnCas12a (C), and AsCas12a (D) (n = 3). The plotted data represent Fa fluorescence values measured every 10 minutes over a total duration of 2 hours. *(ns: non-significant; **p < 0.01; ***p < 0.001).*

(E-G) Energy transfer from the Fd to the Fa was evaluated in the group “C” ssDNA with 1 to 4 sites of 5mC modification, using LbCas12a (E), FnCas12a (F), and AsCas12a (G) (n = 3). The plotted data represent Fa fluorescence values measured every 10 minutes over a total duration of 2 hours. *(ns: non-significant; ***p < 0.001).*

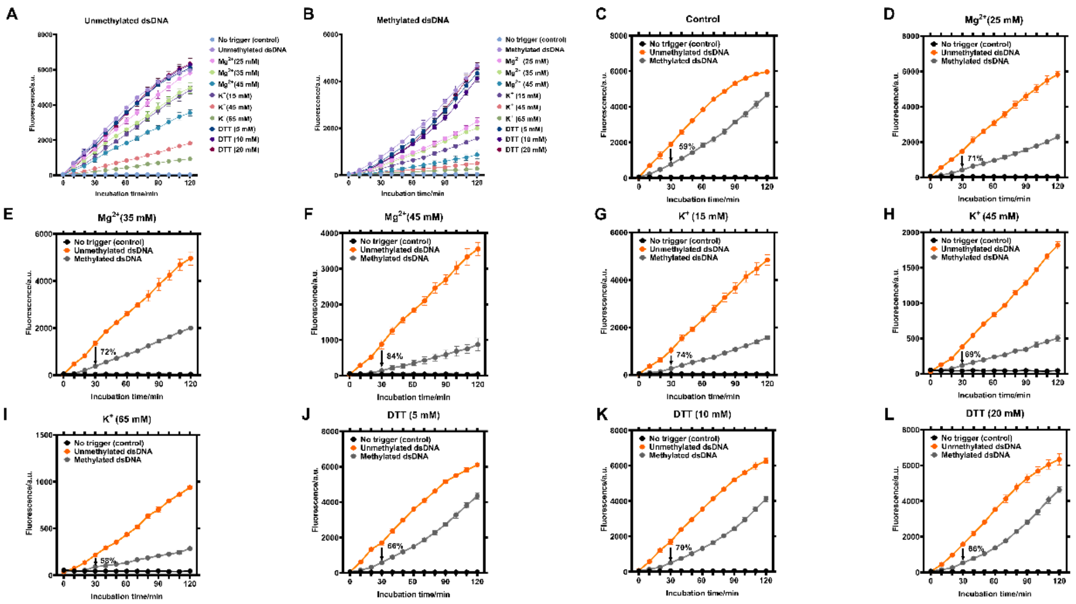

Supplementary Figure 6.

Optimization of FnCas12a conditions for enhanced discrimination of 5mC-Modified DNA

(A-B) Time-course fluorescence analysis of FnCas12a *trans*-cleavage activity triggered by (A) unmethylated and (B) fully methylated dsDNA under various reaction conditions. The effects of different concentrations of Mg^{2+} (25, 35, and 45 mM), K^{+} (15, 45, and 65 mM), and DTT (5, 10, and 20 mM) on *trans*-cleavage efficiency were evaluated (n = 3). The DNA triggers are the unmodified and 5mC-modified 3CG target dsDNA sequences.

(C-L) The percentage labels indicate the signal reduction caused by DNA methylation at 30 minutes: (C) Control (no additive): 59% reduction. (D) Mg^{2+} 25 mM: 71% reduction. (E) Mg^{2+} 35 mM: 72% reduction. (F) Mg^{2+} 45 mM: 84% reduction. (G) K^{+} 15 mM: 74% reduction. (H) K^{+} 45 mM: 69% reduction. (I) K^{+} 65 mM: 58% reduction. (J) DTT 5 mM: 66% reduction. (K) DTT 10 mM: 70% reduction. (L) DTT 20 mM: 66% reduction (n = 3).

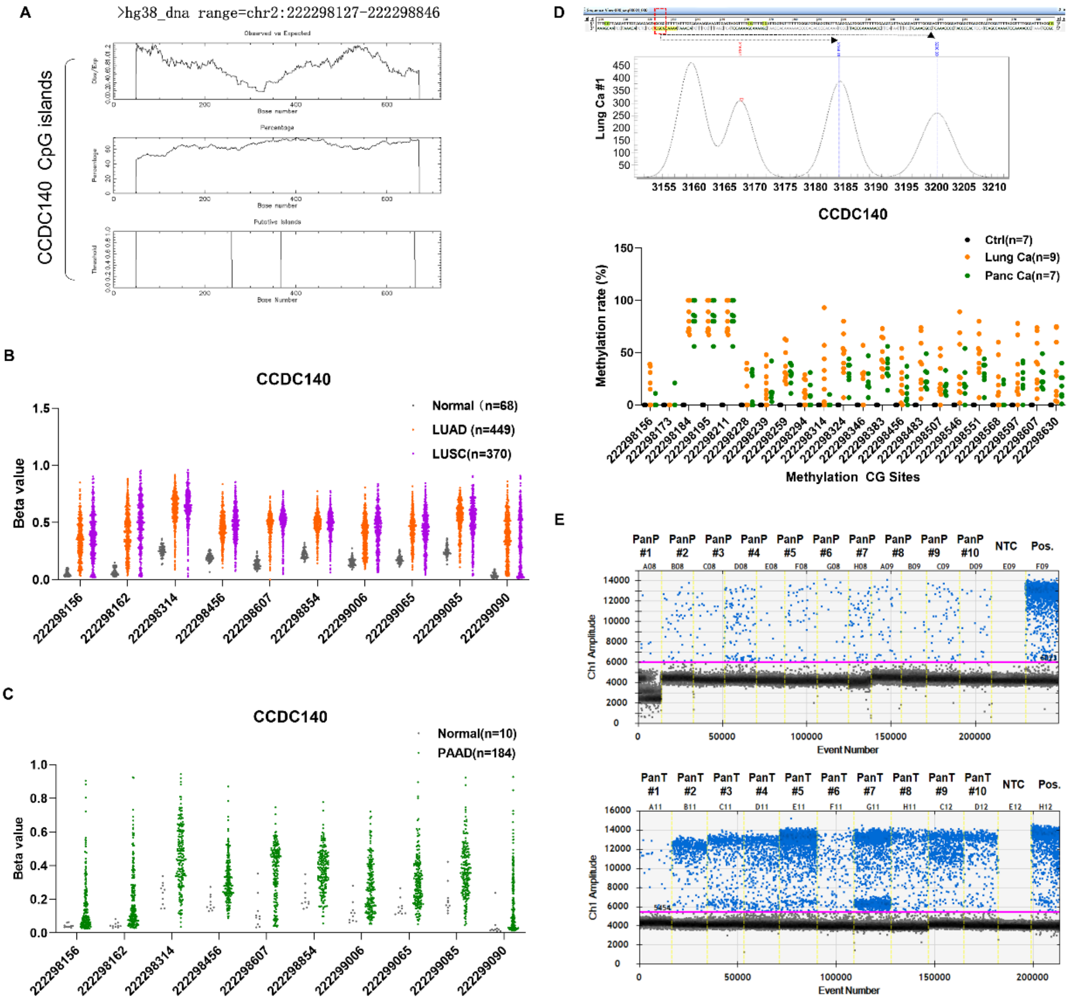

Supplementary Figure 7.

CpG Islands and methylation status of the CCDC140 promoter

(A) CpG islands in the CCDC140 promoter predicted by EMBOSS CpGplot (https://emboss.bioinformatics).

(B) CCDC140 promoter methylation in LUAD (n = 449), LUSC (n = 370), and healthy controls (n = 68), analyzed with SMART (http://www.bioinfo-zs.com/smartapp/). Beta-values show hypermethylation in tumors compared with normal tissue.

(C) CCDC140 promoter methylation in PAAD (n = 184) and healthy controls (n = 10), analyzed with SMART (http://www.bioinfo-zs.com/smartapp/). Beta-values show hypermethylation in tumors compared with normal tissue.

(D) CpG methylation near the CCDC140 promoter analyzed by MALDI-TOF in controls (n = 7), lung cancer (n = 9), and pancreatic cancer (n = 7).

(E) Detection of CCDC140 promoter methylation in pancreatic cancer tissues (n = 10) and matched adjacent non-tumor tissues (n = 10) by ddPCR.

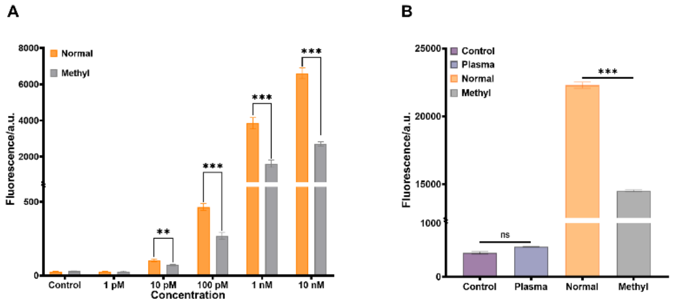

Supplementary Figure 8.

Single gRNA LOD and plasma background assay

(A) Fluorescence signals increased proportionally with dsDNA concentrations ranging from 1 pM to 10 nM, with a LOD as low as 10 pM. Orange bars represent unmethylated 3CG dsDNA (Normal); grey bars represent methylated 3CG dsDNA (Methyl) (n = 3). *(ns: non-significant; **p < 0.01; ***p < 0.001).*

(B) Cas12a reactions were performed to evaluate plasma background interference. The assay included four groups: control, DNA-free plasma, unmethylated synthetic CCDC140 dsDNA, and methylated synthetic CCDC140 dsDNA (n = 2). *(ns: non-significant; ***p < 0.001).*
